# Omega-3 Fatty Acids for the Treatment of Bipolar Disorder Symptoms: A Narrative Review of the Current Clinical Evidence

**DOI:** 10.3390/md23020084

**Published:** 2025-02-15

**Authors:** Evmorfia Psara, Sousana K. Papadopoulou, Maria Mentzelou, Gavriela Voulgaridou, Theophanis Vorvolakos, Thomas Apostolou, Constantinos Giaginis

**Affiliations:** 1 Department of Food Science and Nutrition, School of the Environment, University of the Aegean, 81400 Myrina, Lemnos, Greece; fnsd21013@fns.aegean.gr (E.P.); maria.mentzelou@hotmail.com (M.M.); 2 Department of Nutritional Sciences and Dietetics, School of Health Sciences, International Hellenic University, 57400 Thessaloniki, Greece; souzpapa@gmail.com (S.K.P.); gabivoulg@gmail.com (G.V.); 3 Department of Psychiatry, School of Medicine, Democritus University of Thrace, 68100 Alexandroupolis, Greece; tvorvola@med.duth.gr; 4 Department of Physiotherapy, School of Health Sciences, International Hellenic University, 57400 Thessaloniki, Greece; apostolouthomas@hotmail.com

**Keywords:** bipolar disorder, omega-3, polyunsaturated fatty acids, depressive episodes, maniac episodes, clinical trials, eicosapentaenoic acid, docosahexaenoic acid (DHA), fish, seafoods, drug design

## Abstract

Background: Bipolar disorder is a chronic mental disease that is characterized by depressive and manic episodes. Antipsychotics and mood stabilizers are known therapies that work, but their restrictions and disadvantages resulted in the need for complementary and alternative therapies, such as natural compounds. Omega-3 fatty acids, as basic ingredients of fishes and seafood, play crucial roles in brain development, function of brain membrane enzymes, learning, and many other instances, and their deficiency has been associated with many mental diseases, including bipolar disorder. Methods: The present narrative review aims to critically summarize and scrutinize the available clinical studies on the use of omega-3 fatty acids in the management and co-treatment of bipolar disorder episodes and symptoms. For this purpose, a thorough and in-depth search was performed in the most accurate scientific databases, e.g., PubMed., Scopus, Web of Science, Cochrane, Embase, and Google Scholar, applying effective and relevant keywords. Results: There are currently several clinical studies that assessed the effect of omega-3 fatty acids on the severity of BD symptoms. Some of them supported evidence for the potential beneficial impact of omega-3 fatty acids supplementation in the prevention and/or co-treatment of bipolar disorder severity and symptomatology. Nevertheless, a considerable number of clinical studies did not show high efficiency, rendering the existing data rather conflicting. The above may be ascribed to the fact that there is a high heterogeneity amongst the available clinical studies concerning the dosage, the administration duration, the combination of fatty acids administration, the method designs and protocols, and the study populations. Conclusion: Although the currently available clinical evidence seems promising, it is highly recommended to accomplish larger, long-term, randomized, double-blind, controlled clinical trials with a prospective design in order to derive conclusive results as to whether omega-fatty acids could act as a co-treatment agent or even as protective factors against bipolar disorder symptomatology. Drug design strategies could be developed to derive novel synthetic omega-3 fatty acids analogs, which could be tested for their potential to attenuate the severity of BD episodes and symptoms.

## 1. Introduction

Bipolar disorder (BD) is a mood disorder with intermittent periods of depression and mania or hypomania. BD constitutes a common and recurrent psychiatric disorder marked by a high prevalence, frequent relapses, substantial disability rates, elevated mortality rates, and notable comorbidity [1]. BD affects approximately 2.4% of the world’s population and is associated with reduced functioning, cognitive impairment, and decreased quality of life as a lifelong and especially recurrent disorder [2]. BD represents a substantial problem in both individuals’ lives and healthcare systems, affecting about one to two percent of the population globally [3]. BD is a common and debilitating disorder in which several aspects of the personal, social, and occupational life and interpersonal relationships of patients are negatively affected [3,4]. Numerous factors, including genetic, neurobiological, personality, and environmental factors, can play a role in causing this disease [4,5]. The impact of BD is long-lasting and wide-ranging, affecting not only mental and physical health but also the social and occupational aspects of an individual’s life [6,7]. The WHO highlights that BD ranks among the major mental health contributors to global disability, primarily due to its impact on a young and economically active population [2,8].

The pathophysiology of BD is multifaceted, relating neurotransmitter dysregulation, especially dopamine, serotonin, and glutamate systems [9,10]; oxidative stress; mitochondrial dysfunction; and inflammation [11,12,13]. These pathways are possible therapeutic targets for pharmacological agents and natural compounds [14]. Notably, in recent BD research, the focus was on inflammation, oxidative stress, and mitochondrial dysfunction [10,15]. When focusing on these areas, mood can be stabilized, manic and depressive episodes can be reduced, and long-term outcomes may be improved [16]. The treatment of BD is primarily pharmacological and should be adapted to each phase of the disorder: mania/hypomania, depression, or maintenance [17]. Conventional treatments, such as mood stabilizers like lithium, valproate, and lamotrigine, along with second-generation antipsychotics like quetiapine, play a crucial role in BD management [18]. The treatment’s aim is to improve current symptoms and prevent new episodes. A common challenge is the low patient adherence to treatment, especially throughout manic episodes when insight into the disease is compromised [19]. However, specific medications frequently lead to opposing effects like increased body weight, metabolic problems, and long-term toxicity [9].

Consequently, there is a rising research interest in searching for complementary approaches, like natural products, which may offer mood stabilization and neuroprotective effects with fewer side effects. Recently, there has been a rising interest in the management of psychiatric disorders, including BD, using natural products, such as herbal extracts, phytochemicals, and nutritional supplements [20]. Natural products that are produced from plants and other natural sources, such as fish and seafood, have generally been used in traditional medicine for their psychotropic properties [21]. Recent research has started to explain how these products act, including modulation of neuroinflammatory pathways, oxidative stress, neurotransmitter systems, and the gut–brain axis pathways implicated in the pathophysiology of BD [22]. Several natural compounds, such as omega-3 fatty acids, curcumin, trace elements, flavonoids, and N-acetylcysteine (NAC), have shown promising results mainly in preclinical and less in clinical studies, offering potential benefits as adjunctive treatments to conventional BD therapies [23].

Epidemiological and biochemical studies have demonstrated convincing evidence regarding the relationship between BD and reduced consumption of omega-3 fatty acids, a category of natural compounds that widely exists in fish and seafood [24]. In general, low levels of omega-3 fatty acids in the blood and brain tissue after death have been found in patients with BD. Although there is no clear explanation yet, the lack of omega-3 consumption is considered one of the preventable risk factors for recurrent mood disorders [25]. Fatty acids play various physiological roles in organisms; they are crucial for the structure of cell membranes, metabolic processes, the transmission of nerve impulses, and brain functions [26,27]. Omega-3 fatty acids are influential for brain development, function of brain membrane enzymes, learning, and many other instances, and their deficiency is associated with many psychological cognitive disorders [26,27].

**Figure 1:**
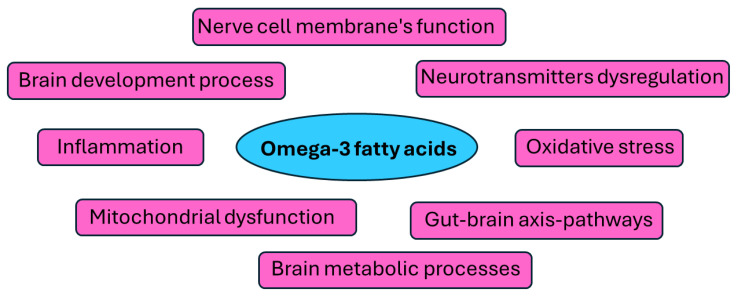
The basic molecular mechanisms through which omega-3 fatty acids may affect the nervous system, BD progression, and symptomatology.

Omega-3 polyunsaturated fatty acids (PUFAs), especially eicosapentaenoic acid (EPA) and docosahexaenoic acid (DHA), have been widely studied for their impact on inflammation, brain health, and neurotransmitter function [28]. They are thought to reduce neuroinflammation and modulate serotonin and dopamine activity, potentially stabilizing mood [29]. Omega-3 fatty acids have been considered to inhibit neuronal signal transduction pathways in a manner similar to that of lithium carbonate and valproate, the two most effective treatments for BD [30,31]. Biochemical studies on human white blood cells indicate that treatment with high-dose omega-3 fatty acids causes the internalization of these polyunsaturated combinations into membrane phospholipids that are necessary for cell signaling [30]. Omega-3 fatty acids have few side effects, mainly gastrointestinal discomfort at high doses. In this respect, several clinical studies have examined the effectiveness of omega-3 fatty acids in BD so far [30,31]. The basic molecular mechanisms through which omega-3 fatty acids may affect the nerve system and BD progression, as well as symptomatology, are depicted in Figure 1.

In this aspect, the aim of our narrative review was to critically summarize and scrutinize the currently existing clinical human studies concerning the potential beneficial effects of omega-3 fatty acids consumption on BD. By critically assessing the existing literature and identifying key areas for future research, we aim to contribute to the current body of knowledge surrounding the effectiveness of omega-3 fatty acids in the clinical practice for the management and co-treatment of BD symptoms.

## 2. Methods

A comprehensive and detailed narrative review was carried out by searching the most reliable scientific databases, e.g., PubMed, Scopus, Cochrane, Embase, Web of Science, and Google Scholar, applying effective and relevant keywords such as “bipolar disorder and omega-3 fatty acids, “bipolar disorder and polyunsaturated fatty acids”, “bipolar disorder and eicosapentaenoic acid”, “bipolar disorder and docosahexaenoic acid”, “psychiatric disease and omega-3 fatty acids”, “psychiatric disease and polyunsaturated fatty acids”, “psychiatric disease and eicosapentaenoic acid”, “psychiatric disease and docosahexaenoic acid”, “omega-3 fatty acids and mental disorders”, “omega-3 fatty acids and mood disorders”, “omega-3 fatty acids and psychiatric diseases”, “omega-3 fatty acids and neurobiological effects”, “omega-3 fatty acids and clinical trials”, “bipolar mania”, “bipolar depression”. We retrieved all clinical studies in humans that are currently available in the international literature and that were written in the English language. Exclusion criteria were experimental in vitro studies, animal in vivo studies, and studies not written in the English language. Gray literature, editorial texts, commentaries, letters to the editor, abstracts in congress records, and articles in non-peer-reviewed journals were also excluded from the final analysis.

**Figure 2:**
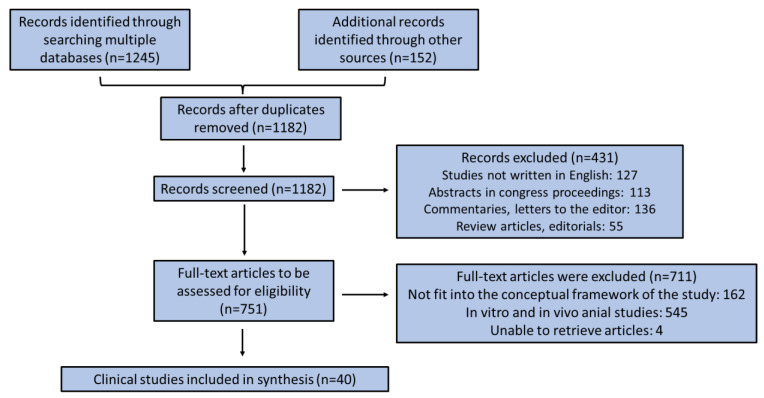
Flow chart diagram for studies selection and enrollment.

The search was supplemented by checking the reference lists of relevant clinical studies and manually searching the reference lists of key journals, commentaries, editorials, and abstracts in congress records. The retrieved studies were carefully searched for relevant surveys included in their manuscript. There was no time limitation concerning the included studies retrieved by the scientific databases. All authors acted as reviewers. To enhance the consistency among reviewers, all of them screened the retrieved publications, discussed the results, and amended the screening and data extraction manual before initiating screening for this review. They alternately assessed the titles, abstracts, and full texts of all publications retrieved from their searches for potentially relevant publications with good methodology and credible study design. We resolved disagreements on study selection and data extraction by consensus and discussion with all the authors/reviewers if required. The findings were selected based on relevance, and the most relevant ones were chosen and mentioned below according to the flow chart diagram depicted in Figure 2.

## 3. Results

### 3.1. The First Clinical Studies During the Decade 1999–2009

The clinical studies evaluating the role of omega-3 fatty acids in bipolar disorder symptoms are summarized in Table 1. The clinical research on the effect of omega-3 fatty acids against BD symptoms began 25 years ago by Stoll et al., who examined whether omega-3 fatty acids could exert mood-stabilizing properties in BD [32]. This study implemented a 4-month, double-blind, placebo-controlled pilot design to compare omega-3 fatty acids (9.6 g/day) vs. placebo (olive oil), in addition to the standard treatment in 30 BD patients. The patients of the intervention group received seven capsules twice daily for a total daily omega-3 fatty acids dosage of 6.2 g of EPA and 3.4 g of DHA [32]. A Kaplan–Meier survival analysis of the cohort revealed that the omega-3 fatty acids patient group exhibited a substantially longer period of remission compared to the placebo group. In addition, for nearly every other secondary outcome measure, the omega-3 fatty acid group performed better than the placebo group [32]. Omega-3 fatty acids treatment was generally well tolerated without severe adverse effects and improved the short-term course of disease in this preliminary study [32]. However, the baseline clinical state of the enrolled patients cannot permit an assessment of the antimanic effects of omega-3 fatty acids [32]. Moreover, although this study was a double-blind, placebo-controlled clinical trial, several methodological factors must be considered. More importantly, this study included a mixture of BD types I and II, varied mood states at its entry, and varying concomitant medications, rendering its design less accurate than in an ideal clinical trial [32].

Four years later, in adults, a double-blind, randomized controlled study of EPA monotherapy was performed (unlike the previous study by Stoll et al. in which EPA was added to the existing therapy), which failed to show efficacy [33]. In this aspect, 6 g of EPA daily uptake was compared with placebo for 4 months in the treatment of either acute depression or rapid cycling in adults with BD [33]. This study investigated the assumption of whether there could be a depletion of omega-3 fatty acids in erythrocyte membranes in patients with BD. Fatty acid compositions of erythrocyte membranes in 20 bipolar manic patients and 20 healthy controls were analyzed. Arachidonic acid and DHA compositions were substantially lowered in BD patients compared to healthy controls. There were no differences in total omega-3 and omega-6 fatty acids. This discrepancy was ascribed to potential mechanisms of action of mood stabilizers in patients with BD [33].

Epidemiologic data have linked fish consumption and omega-3 fatty acids absorption with the prevalence of BD at a national level. For this purpose, a cross-national study assessed lifetime prevalence rates in various countries for BD-I, BD-II, and bipolar spectrum disorder [34]. These epidemiological studies applied structured diagnostic interviews with analogous diagnostic criteria and were population-based with large sample sizes. The lifetime prevalence rates (number per 100,000) of BDs and schizophrenia were standardized at each site, and a design weight was estimated for each subject, stratified for age and sex. Prevalence rates weighted in this manner provided estimates of whether each site experienced the same age and sex distributions. The lifetime incidence rate for bipolar spectrum disorder ranged across 12 countries from 0.2 in Iceland to 6.5 in Germany. The lifetime incidence rate for BD-I varied across 11 countries, from 0.3 in Taiwan to 2.6 in Israel [34]. The lifetime prevalence rate for BD-II varied across eight countries, from 0.1 in Taiwan to 2.0 in Hungary. Simple exponential decay regressions showed that greater seafood consumption predicted lower lifetime frequency rates of BD-I, BD-II, and bipolar spectrum disorder [34].

Moreover, according to the above study, BD-II and bipolar spectrum disorder exhibited an apparent vulnerability threshold below 50 lb of seafood/person/year [34]. The level of 50 lb per person per year was, at most, approximately 300 g of seafood per person per day. Assuming that 100 g of seafood contains 1 g of EPA plus DHA (U.S. Department of Agriculture food tables), this level corresponds to a maximum consumption of 3 g of EPA plus DHA per person daily [34]. The strongest association was found between BD-II and seafood and fish consumption, which was in agreement with the observation that omega-3 fatty acids were more effective in reducing depressive than manic symptoms [32]. These data described a strong relationship between greater seafood consumption and lower incidence rates of BDs. These data provided a cross-national context for understanding ongoing clinical intervention trials of omega-3 fatty acids in BDs [34]. Although these findings did not demonstrate a causal relationship, they were consistent with the assumption that an inadequate dietary intake of essential omega-3 fatty acids may increase the risk of affective disorders. A limitation of this study was that it could not determine whether low seafood consumption can increase lifetime risk for BDs because of effects solely in adulthood or due to dietary insufficiency in early neurological development or both of them [34].

One year later, Hirashima et al. assumed that alterations in brain membrane composition resulting from omega-3 fatty acid administration in patients with BD may lead to greater membrane fluidity, as detected by reductions in T2 values [35]. Women with BD (*N* = 12) received omega-3 fatty acids for 4 weeks. In fact, the enrolled women received either high doses of omega-3 fatty acids for a total of 5.0–5.2 g/day of EPA, 3.0–3.4 g/day of DHA, and 1.7 g/day of other omega-3 fatty acids (*N* = 6) or low doses of omega-3 fatty acids for a total of 1.3 g/day of EPA acid and 0.7 g/day of DHA (*N* = 6) [35]. A cohort of BD patients (*N* = 9) and a group with healthy individuals (*N* = 12) did not receive omega-3 fatty acids. The Hamilton Depression Rating Scale and the Young Mania Rating Scale were administered and completed by all individuals at baseline and in 4th week [35]. Magnetic resonance imaging (MRI) scans were conducted by using a 1.5 T magnetic resonance scanner and T2 values through a single 5 mm axial plane at the level of the bilateral basal ganglia at baseline and after 4 weeks. T2 is a measure of the rate of dephasing, and therefore, the packing of the lipid molecules and lateral membrane mobility should affect these values. Both the omega-3 fatty acids high-dose and omega-3 fatty acids low-dose groups showed substantial reductions in percentage change in T2 median times compared to the comparison group. Notably, there was a dose-dependent effect when the bipolar omega-3 fatty acids group was subdivided into high- and low-dose cohorts [35]. Hence, omega-3 fatty acids lowered T2 values in agreement with the assumption that the fluidity of cell membranes was altered. On the other hand, there were no significant changes in the rate of depressive or manic episodes in the three groups from baseline to the 4th week [35]. However, there was a modest but nonsignificant association between omega-3 fatty acids-induced reductions in T2 and depressive episode rate in the high-dose fish oil cohort. However, this study had some limitations as it did not detect significant changes in mood states in the BD individuals after omega-3 fatty acids treatment. Thus, it was suggested that although the biological effects of omega-3 fatty acids were detectable within 4 weeks of treatment, the effects on mood state may require a longer time interval [35].

One year later, Osher et al. performed an open-label add-on clinical trial evaluating EPA treatment potential in BD depression [36]. Twelve BD-I outpatients with depressive symptoms diagnosed with DSM-IV were treated with 1.5 to 2 g/day of the omega-3 fatty acid EPA for up to 6 months. Eight of the ten patients who completed at least one month of follow-up accomplished a 50% or greater reduction in depressive episode rate within one month [36]. No patients developed hypomania or manic symptoms, and no significant side effects were reported. However, this study was limited both by the open-label design and by the small sample size. Moreover, the enrolled patients were treated in an outpatient setting, and therefore, the most severely depressed BD patients who require hospitalization were not represented [36].

The same year, Sagduyu et al. carried out a 37-patient continuation study of the open-ended omega-3 fatty acids add-on study [37]. The enrolled BD patients were the original 19 patients, along with 18 new patients recruited and followed in the same manner as the initial 19 patients. A patient’s clinical status was examined using the Clinical Monitoring Form. The enrolled patients reported the frequency and severity of irritability experienced during the former 10 days [37]. The incidence was assessed by the percentage of days in which individuals experienced irritability, and the severity of the irritability was determined on a Likert scale of 1–4. The treatment period ranged from 84 days to 1995 days, with a mean duration of 439.62 days. The irritability component of the Young Mania Rating Scale was also consistently recorded in 13 of the 39 patients [37]. Notably, this study found that patients had persistent irritability despite their ongoing pharmacologic treatment and psychotherapy. Omega-3 fatty acids added to the existing treatment helped with the irritability component of a significant percentage of BD patients with a persistent sign of irritability. This study also revealed that the optimum effective dose for irritability was 1–2 gr of EPA plus DHA per day low dose (1 to 2 g per day) [37].

Afterward, Keck et al. conducted a 4-month, randomized, placebo-controlled, adjunctive clinical trial of ethyl-EPA 6 g/day in the treatment of BD depression and rapid cycling BD [38]. The enrolled individuals were receiving mood-stabilizing medications at therapeutic doses or plasma concentrations. The measures of efficacy were early study discontinuation, changes from baseline in depressive symptoms and in manic symptoms, and manic exacerbations [38]. This study also measured side effects and bleeding time, a biomarker of drug action. Ethyl-EPA was generally well tolerated, and most adverse events were mild. Overall, there were no significant differences in any outcome measure between the ethyl-EPA and placebo groups. Thus, this study did not find overall evidence of efficacy for adjunctive treatment with ethyl-EPA 6 g/day in outpatients with BD depression or rapid cycling BD [38]. The authors suggested that the possible therapeutic effects of EPA may have been masked by coadministration with mood-stabilizing agents [38].

In the same year, Frangou et al. assessed the efficacy of ethyl-EPA in treating depression in BD patients [39]. In a 12-week, double-blind study, individuals with BD depression were randomly assigned to adjunctive treatment with a placebo (*n* = 26) or with 1 g/day (*n* = 24) or 2 g/day (*n* = 25) of ethyl-EPA. This study also did not find considerable benefit of 2 g over 1 g ethyl-EPA daily [39]. Significant improvement was noted with ethyl-EPA treatment compared with placebo concerning the rate of depressive episodes and the Clinical Global Impression Scale scores. Moreover, ethyl-EPA doses were well tolerated. Thus, it was suggested that adjunctive ethyl-EPA may be an effective and well-tolerated intervention in bipolar depression. However, this study did not find overall evidence of efficacy for adjunctive treatment with EPA 6 g/day in outpatients with BD depression or rapid cycling BD [39]. Moreover, this was a small study that only assessed the short-term efficacy and tolerability of ethyl-EPA treatment in BD. It also assessed the efficacy and tolerability of ethyl-EPA as adjunctive treatment in BD and not as monotherapy [39].

In the same year, another randomized, double-blind, placebo-controlled pilot study was conducted at three sites (*N* = 10) comparing an omega-3 fatty acid (DHA) versus placebo and added to psychosocial treatment for pregnant women with BD who choose to discontinue all conventional psychotropic medications while attempting to conceive [40]. In this pilot study, 10 Caucasian women received DHA (2000 mg/day) or a matching placebo for one year while attempting to conceive (BP1 = 9, BPII = 1). All participants had a clinical status of “recovered”, i.e., no more than two moderate mood symptoms, for 6 months prior to study entry. DHA was well tolerated, suggesting that a larger study could be feasible [40]. However, the data of this study were not considered sufficient to support a recommendation of monotherapy treatment as a substitute for standard pharmacologic treatments. On the other hand, participants were enthusiastic about the possibilities of mood-stabilizing benefits in addition to health benefits to both mother and fetus [40].

The next year, Fragou et al. assessed whether ethyl-EPA may be beneficial in the treatment of BD and whether it could exert a neurotrophic/neuroprotective effect in patients with neuropsychiatric disorders [41]. In this aspect, they examined whether ethyl-EPA treatment of BD patients may be associated with enhanced brain levels of N-acetylaspartate, a putative marker of neuronal integrity. Fourteen female BD outpatients with moderate depressive symptoms were administered 2 g of ethyl-EPA per day or placebo for 12 weeks in a randomized, double-blind manner [41]. A significant rise in N-acetylaspartate levels was observed in the ethyl-EPA treatment group compared with the placebo group. These results provided the first evidence for a probable neurotrophic role of ethyl-EPA treatment in BD, highlighting the necessity for a more detailed investigation of its mechanism of action and therapeutic potential [41]. It should be noted that since both the ethyl-EPA and placebo groups were similarly treated with respect to lithium dose and serum levels, it may be questionable whether the observed elevation in N-acetylaspartate could be attributed to lithium treatment. Hence, the possibility of a synergistic action between lithium and ethyl-EPA cannot be excluded. The precise time course of ethyl-EPA-associated N-acetylaspartate alterations was not clear since this study only collected data at two time points. Thus, the results of this study may suggest that such changes could be present at least 3 months after treatment initiation [41].

One year later, another prospective open-label pilot clinical study assessed the effectiveness and safety of omega-3 fatty acids (Omegabrite(R) brand) in the treatment of pediatric BD [42]. Individuals (*N* = 20) were outpatients of both sexes aged 6 to 17 years with a DSM-IV diagnosis of pediatric BD and a Young Mania Rating Scale score of >15. They were treated over an 8-week period in an open-label clinical trial with omega-3 fatty acids 1290–4300 mg combined EPA and DHA. The enrolled patients receiving more than 2.0 g dose showed greater clinical improvement at the endpoint compared to those receiving 2.0 g or less as measured by the change in the Young Mania Rating Scale score [42]. There was an increase in the percentage distribution of EPA relative to total fatty acids in plasma and in RBC membranes. There was also an increase in the percentage distribution of red blood cell (RBC) membrane DHA. Participants experienced a significant but modest 8.9+/−2.9-point reduction in the rate of manic episodes. Although this indicated significant improvement, individuals continued to have clinically significant residual symptoms of mania. Likewise, although there was a significant 10.4 ± 9.9-point reduction in Child Depression Rating Scales scores, the group mean at the endpoint indicated continued symptoms of depression. Adverse events were few and mild [42].

The above study also showed that RBC membrane levels of EPA and DHA increased in treated individuals [42]. As only 35% of these individuals exhibited a response by the usually accepted criteria of >50% decrease on the Young Mania Rating Scale score, omega-3 fatty acids treatment was associated with a very modest improvement in manic symptoms in children with pediatric BD [42]. However, this study had some limitations as it was an open-label and uncontrolled clinical trial, and its results should be considered preliminary. Because it was an open study, assessments were not blind to treatment status. Despite these considerations, results from this prospective, open study of monotherapy with omega-3 fatty acids in the over-the-counter product Omegabrite^®^ suggested that manic symptoms could be rapidly reduced in youths with BD with a safe and well-tolerated nutritional supplement [42]. It should also be noted that some studies, such as those of Stroll et al. and Keck et al., utilized high doses of omega-3 fatty acids (9.6 g EPA daily and 6 g EPA daily, respectively) with no significant effects [32,38]. In contrast, the lowest daily dose (1 g EPA) was more useful than a placebo or supplementing with either 2 g or 4 g of EPA in the adjunctive treatment of depressed adults [43]. These findings could suggest that omega-3 fatty acids may be most effective in a lower dosing range, supporting evidence that higher doses may be ineffective.

Furthermore, another clinical study showed that manic symptom severity was negatively associated with levels of free EPA. In fact, Sublette et al. performed a pilot study to compare plasma levels of free (non-esterified) and esterified PUFAs between patients in an acute manic episode and healthy volunteers [44]. They aimed to investigate potential relationships between symptom severity and levels of omega-3 fatty acids and of the arachidonic acid metabolite, prostaglandin E2. In fact, patients (*n* = 10) who were medication-free for at least two weeks and requesting inpatient admission for an acute manic episode were compared with healthy volunteers (*n* = 10) [44]. Symptom severity was assessed at admission and after 6 weeks of real treatment. Fasting baseline free and esterified plasma levels of DHA, EPA, arachidonic acid, and prostaglandin E2 were determined. Prostaglandin E2 levels were tested again in 6 weeks. However, no differences between groups were found in individuals’ levels or total omega-3 fatty acids or prostaglandin E2. Notably, among the participants, manic symptom severity was negatively associated with levels of free arachidonic acid and free EPA and positively with the free arachidonic acid/EPA ratio. On the other hand, prostaglandin E2 levels did not differ between groups or in subjects pre- and post-treatment [44].

One year later, 15 children and adolescents (9–18 years) diagnosed with juvenile BD (JBD) and 15 healthy age and sex-matched controls were assessed for dietary intake and fasting RBC membrane concentrations of omega-3 fatty acids [45]. Participants with JBD were diagnosed with BD-I (*n* = 7), BD-II (*n* = 3), or BD not otherwise specified (NOS, *n* = 5), and all received mood-stabilizing medication. However, RBC membrane concentrations of EPA and DHA were not significantly lower in participants diagnosed with JBD compared with healthy individuals after adjusting for dietary intake [45]. Moreover, RBC EPA and DHA levels were not considerably different among participants, with JBD receiving either lithium or valproate as their primary mood-stabilizing medication. Notably, RBC DHA was negatively related to clinician ratings of depression using the Clinical Global Impressions scale and participant ratings of aggression using the Beck Youth Inventories [45]. Plasma EPA%, which reflects recent dietary intake, was also positively related to clinician ratings of mania, parent ratings of externalizing symptoms, and participant ratings of disruptive behavior. On the other hand, RBC EPA was not considerably related to any psychological measures. Although lower RBC concentrations of omega-3 fatty acids were ascribed to lower dietary intakes in this study, previous evidence had linked reduced omega-3 fatty acids to the etiology of BD. As RBC DHA was also negatively related to symptoms of depression, a randomized placebo-controlled study exploring omega-3 fatty acids supplementation as an adjunct to standard pharmacotherapy was highly recommended in this patient population [45]. In addition, blood concentrations were related to omega-3 fatty acids intake, and they were not substantially different between participants with JBD and controls after adjusting for intake. Thus, the data from this study suggested that there could not be a genetic abnormality in omega-3 fatty acids incorporation into RBC in participants with JBD. It should be noted that the results of this study should be treated with caution and require replication due to the small number of participants. However, given the significant relationship between intake and RBC membrane concentrations of omega-3 fatty acids, the methods used in this study appear to be valid [45].

In the same year, McNamara et al. determined the omega-3 fatty acid composition of the postmortem orbitofrontal cortex (OFC, Brodmann area 10) of patients with BD (*n* = 18) and age-matched healthy individuals (*n* = 19) [46]. After correction for multiple comparisons, DHA (−24%), arachidonic acid (−14%), and stearic acid (−4.5%) compositions were significantly lower, and cis-vaccenic acid (+12.5%) composition was considerably higher in the OFC of BD patients compared to normal controls [46]. OFC DHA and arachidonic acid deficits were higher in patients plus normal controls with high versus low alcohol abuse severity. These results added to a growing body of evidence implicating omega-3 fatty acids deficiency as well as the OFC in the pathoaetiology of BD [46]. BD patients who died by suicide also exhibited selective deficits in OFC DHA composition, a finding that was consistent with previous studies, suggesting that omega-3 fatty acid insufficiency may increase the risk of suicide [46].

The same research group of Clayton et al. investigated the efficiency of omega-3 fatty acids supplementation in the treatment of mania and depression in JBD when given as an adjunct to standard medication treatment [47]. In fact, 15 children and adolescents with JBD received 360 mg per day EPA and 1560 mg per day DHA for 6 weeks in an open-label study. Intake and fasting RBC omega-3 fatty acids, mania, depression, and global function were assessed before and after intervention [47]. It was found that RBC EPA and DHA were considerably higher after intervention. Clinician ratings of mania and depression were substantially lower, and global functioning was considerably higher after intervention. Parent ratings of internalizing and externalizing behaviors were also considerably lower [47]. However, due to the small number of participants, the authors suggested that larger randomized controlled clinical trials were required in this study population in order for more precise conclusions to be drawn [47].

Another case–control study by McNamara et al. determined erythrocyte fatty acids composition in healthy adult male and female individuals without a history of psychiatric disease (*n* = 20) and medication-free patients with major depressive disorder (MDD) (*n* = 20) or BD (*n* = 20) [48]. They used medication-free patients because preclinical evidence had suggested that antidepressant and mood-stabilizer medications can change membrane fatty acid turnover and/or biosynthesis [49,50] and may confound associations between fatty acid status and symptom severity scores. It should also be noted that dietary fish or fish oil intake was linearly correlated with erythrocyte EPA + DHA levels [51,52]. Moreover, fish oil supplementation was sufficient to enhance erythrocyte EPA + DHA levels in pediatric and adolescent patients with BD, as also shown in the studies of Wozniak et al. and Clayton et al. [42,47]. While it is not known whether bipolar families consume fish less frequently than the general population, emerging evidence suggests that patients with mood disorders may have poorer nutritional habits [53,54,55].

### 3.2. Clinical Studies During the Decade 2010–2019

McNamara et al. evaluated the effects of gender because gonadal hormones have a considerable influence on omega-3 fatty acids biosynthesis and erythrocyte composition [48]. In this study, both MDD (−20%) and BD (−32%) patients exhibited considerably lower erythrocyte DHA composition compared to healthy individuals, and there was a trend for lower DHA in BD patients compared to MDD patients (−15%) [48]. There were no gender differences for DHA in any group. Other omega-3 fatty acids, including EPA and docosapentanoic acid, were not different. Erythrocyte DHA composition was inversely associated with indices of delta-9 desaturase activity and with elevations in oleic acid composition and delta-6 desaturase activity [48]. On the other hand, erythrocyte DHA composition was not substantially associated with depression or mania symptom severity scores [48]. The above data suggested that a defect in peroxisome function may contribute to erythrocyte DHA deficits in MDD and BD. If this assumption is true, this could have important implications for omega-3 fatty acids interventions aimed at reversing this deficit. However, this study has certain limitations as data regarding diet and lifestyle factors (e.g., cigarette smoking) were not available to evaluate their contribution to the above findings [48].

In the same year, Gracious et al. performed a 16-week randomized, placebo-controlled clinical trial of flax oil containing the omega-3 fatty acid α-linolenic acid in pediatric BD [56]. In fact, children and adolescents aged 6–17 years with symptomatic BD-I or BD-II (*n* = 51), manic, hypomanic, mixed, or depressed, were randomized to either flax oil capsules containing 550 mg α-linolenic acid per 1 g or an olive oil placebo adjunctively or as monotherapy. Titrated over 16 weeks. Primary outcomes included changes in the Young Mania Rating Scale, Child Depression Rating Scale—Revised, and Clinical Global Impressions—Bipolar ratings using Kaplan–Meier survival analyses [56]. This study found that there were no significant differences in primary outcome measures when compared to treatment assignment. However, clinician-rated Global Symptom Severity was negatively associated with final serum omega-3 fatty acids compositions: % α-linolenic acid, % EPA, and positively associated with final DHA. The mean duration of treatment for α-linolenic acid was 11.8 weeks versus 8 weeks for placebo. However, the longer treatment duration for α-linolenic acid was not significant after adjusting for baseline variables [56].

In the same study, when data were analyzed by reassigning groups based on meaningful change in EPA levels, only 8 of 34 (24%) participants were considered to have adequate exposure to flax oil [56]. In this group, there was clinician-rated evidence of response, with improvement in overall disease and mania. Producing this change in EPA levels required a dose of 10–12 capsules per day, and most of the participants were either unable to comply or had a variant in fatty acid desaturase enzyme production, reducing conversion of α-linolenic acid to EPA [56]. Thus, it was speculated that persistence with similar regimens in future studies could not be practical. The authors supported the idea that studies of essential fatty acid supplementation may be feasible and well-tolerated in the pediatric population. Although flax oil may decrease disease severity in children and adolescents with BD who have meaningful increases in serum EPA percent levels and/or decreased DHA, individual variations in the conversion of α-linolenic acid to EPA, as well as dosing burden, favor the use of fish oil both for clinical trials and clinical practice [56].

The above 16-week follow-up clinical trial was much longer than typical acute trials and may be associated with ‘study fatigue’ given the possibility of low compliance rates with capsule dosage [56]. However, this may not have an adequate length of follow-up to evaluate the long-term risks and clinical benefits of flax oil usage. In all three survival models, the length of follow-up was too short to allow estimation of the median survival time for the flax oil group [56]. Another limitation of this study was that many of the secondary analyses were based on small numbers of cases. A third limitation was that participants in the trial were predominantly white and middle-class. It is important to consider when generalizing these findings to other settings, not only because of differences or similarities in culture but also because genetic polymorphisms could potentially result in varying efficacy for flax oil augmentation [56].

Two years later, another double-blind, randomized add-on clinical trial aimed to assess the effects of omega-3 fatty acids, given as fish oil capsules, with and without oral cytidine, a pyrimidine with reported preclinical and clinical antidepressant-like effects, in patients with BD [57]. A total of 45 outpatients with diagnosed BD-I were recruited for this 4-month clinical study. Treatment groups were (1) oral cytidine + omega-3 fatty acids, (2) placebo + omega-3 fatty acids, and (3) placebo + placebo control [57]. Fish oil was received as four capsules twice a day (approximately 3 g of EPA/day), and cytidine was administered as four capsules twice daily (2 g cytidine/day). Clinical measures improved in all treatment groups. However, there were no significant differences between groups, including the change in probability of symptoms of depression or mania, change in positive ratings of depression or mania, or change in Global Assessment of Functioning scores [57]. Neither cytidine + omega-3 fatty acids nor placebo + omega-3 fatty acids treatment was superior to placebo treatment. Despite the preclinical studies suggesting that the effect of omega-3 fatty acids may be enhanced with pyrimidines, add-on cytidine did not substantially improve mood symptoms in BD. In addition, although a power analysis indicated that the sample size could be adequate to see beneficial effects similar to those previously reported, omega-3 fatty acids treatment by itself was not superior to placebo for BD [57].

One year later, a clinical study determined whether such omega-3 fatty acids deficit may exist in patients with BD and to characterize the overall plasma fatty acid profile in these patients [58]. Fasting plasma levels of 15 fatty acids in 42 patients diagnosed with BD in a non-acute phase and in 57 age- and gender-matched healthy individuals were determined. This study found that plasma DHA levels were substantially decreased in BD patients [58]. Compared with healthy individuals, patients exhibited greater plasma levels of all other fatty acids, including arachidonic acid, alpha-linolenic acid, and EPA. Although this study observed substantial DHA deficits in the plasma of BD patients, these findings cannot support the therapeutic use of alpha-linolenic acid and/or EPA supplementation [58]. The authors suggested that DHA may provide a basis for possible pharmacological intervention in psychiatric disorders at the level of second messengers linked to the phosphatidylinositol cycle. Moreover, measurement of fatty acid levels in plasma appeared to be more reliable and reproducible compared to assays of erythrocyte fatty acid content [58].

Two years later, Wozniak et al. performed a pilot 12-week, randomized, double-blind, controlled clinical trial to evaluate the efficiency and tolerability of the EPA/DHA omega-3 fatty acids and inositol as monotherapy and in combination in children 5–12 years of age with BD spectrum disorders [59]. The enrolled children were randomized to one of three treatment arms: inositol plus placebo, omega-3 fatty acids plus placebo, and the combined active treatment of omega-3 fatty acids plus inositol. Children weighing ≥25 kg received 2000 mg (4 × 500 mg capsules) of inositol or placebo daily, and those weighing <25 kg received 80 mg per kg rounded down to the nearest 500 mg capsule [59]. All participants also received 975 mg EPA daily, with six of the soft gel capsules, which was 1650 mg combined EPA + DHA total daily dose, or six matched placebo capsules daily. This dose was maintained for the duration of the trial and was permitted to be separated into two daily doses [59]. Twenty-four children were exposed to treatment (≥ 1 week of study completed) (inositol [*n* = 7], omega-3 fatty acids [*n* = 7], and omega-3 fatty acids plus inositol [*n* = 10]). Fifty-four percent of the children finally completed the study. Children randomized to the omega-3 fatty acids plus inositol arm had the largest score decrease compared to the improvement from baseline to end point concerning the Young Mania Rating Scale. Similar results were found for the Children’s Depression Rating Scale and the Brief Psychiatric Rating Scale [59]. Thus, this study supported evidence that the combined treatment of omega-3 fatty acids plus inositol can attenuate the symptoms of mania and depression in prepubertal children with mild to moderate bipolar spectrum disorders. The above findings should be interpreted taking into account some limitations, which include the exclusion of severely ill children, a 54% completion rate, and a small sample size [59].

Moreover, Fristad et al. performed a pilot clinical study to evaluate the efficacy of omega-3 fatty acids supplementation, the individual family psychoeducational psychotherapy (IF-PEP), and their combination in youth with subsyndromal BDs (bipolar disorder not otherwise specified [BD-NOS] or cyclothymic disorder) [60]. More to the point, they accomplished a 12-week, randomized trial of omega-3 versus placebo and IF-PEP versus active monitoring (AM) using a 2 × 2 design (omega-3 + PEP: *n* = 5; omega-3 fatty acids + AM: *n* = 5; placebo + PEP: *n* = 7; placebo + AM: *n* = 6) [60]. Twenty-three youth at the age of 7–14 years with BP-NOS or cyclothymic disorder were enrolled via community advertisements and clinician referrals. Some participants received stable medication for attention-deficit/hyperactivity disorder and sleep aids but no other psychotropics. Independent evaluators assessed participants at screen; baseline; and 2, 4, 6, 9, and 12 weeks [60]. Primary outcome measures were the Kiddie Schedule for Affective Disorders (K-SADS), Depression (KDRS), and Mania (KMRS) Rating Scales, Children’s Depression Rating Scale-Revised, and Young Mania Rating Scale [60]. Omega-3 fatty acids groups received two 500 mg omega-3 fatty acids capsules (350 mg EPA, 50 mg DHA; 100 mg other omega-3) twice daily for a total daily dose of 2000 mg of omega-3 fatty acids (1400 mg EPA, 200 mg DHA; 400 mg other). All participants had a comorbidity. The most frequent comorbid diagnoses were anxiety (83%), attention-deficit/hyperactivity disorder (74%), and disruptive behavior disorders (65%) [60].

In the above study, most participants (83%) completed the 12-week clinical trial. Intent-to-treat analyses indicated substantial improvement in depressive symptoms (KDRS) for combined treatment compared to placebo and AM [60]. Across groups, manic symptoms improved over time without considerable treatment effects. The effect of IF-PEP on child depression compared with AM was medium to large. The effect of omega-3 fatty acids on depression was medium (KDRS). Hence, IF-PEP and omega-3 fatty acids were well tolerated and associated with improved mood symptoms among youth with BP-NOS and cyclothymic disorder [60]. However, the above study had some limitations. It is of note that group differences in depression trajectories were primarily on KDRS scores, a filtered rating of depression, rather than the CDRS-R (which is more likely to capture symptoms of comorbid conditions) [60]. This is possibly ascribed to the fact that PEP was designed to specifically treat mood, while the KDRS is a more precise measure of depression. Moreover, the findings of this study should be interpreted with caution due to its small sample size. In addition, the moderately low baseline manic symptom severity made it difficult to identify differential treatment effects for manic symptoms. Additionally, it is not known what EPA:DHA ratio could be crucial for children or adults with mood disorders. The 7:1 ratio of EPA to DHA used in this study on the recommendation of lipid experts was different from the 2.5:1 ratio naturally found in fish [60].

In the same year, an observational, parallel-group study compared biomarkers between healthy individuals (*n* = 31) and symptomatic patients with BD (*n* = 27) during the disease and after symptomatic recovery (follow-up) [61]. The individuals with BD were mainly diagnosed with BD-I (*n* = 21, 78%) and a smaller number with BD-II (*n* = 6, 22%). Plasma concentrations of five PUFAs [linoleic acid (LA), arachidonic acid (AA), alpha-linolenic acid (ALA), DHA, and EPA], two saturated fatty acids (palmitic acid and stearic acid), and two monounsaturated fatty acids (palmitoleic acid, oleic acid) were measured in esterified and unesterified forms [61]. The measured ratios included UE:E for the five PUFAs, ratios of omega-3 PUFAs (DHA:ALA, EPA:ALA, EPA:DHA), and the ratio of omega-6:omega-3 AA:EPA [61]. This study found that unesterified EPA was lower in BD patients compared to healthy individuals, with a large effect size; however, it was not statistically significant after adjustment for multiple comparisons. Moreover, no substantial difference was found in any plasma PUFA concentration between BD patients and healthy individuals after Bonferroni correction for 40 comparisons. Neither depressive severity nor mania severity was also considerably associated with any PUFA concentration [61]. Exploratory comparison revealed lower unesterified/esterified EPA in BD patients compared to healthy individuals. At follow-up in the BD group, unesterified, esterified E DHA:ALA, and unesterified EPA:ALA were lowered. Exploratory correlations of clinical variables also revealed that mania severity and suicidality were positively associated with the unesterified/esterified EPA ratio and that several plasma levels and ratios were related to panic disorder and psychosis [61].

Concerning the above study, a large effect size of decreased unesterified EPA and a lower plasma unesterified/esterified concentration ratio of EPA in the symptomatic BD state could be an important factor in vulnerability to a mood state [61]. Altered omega-3 fatty acid ratios could also indicate changes in omega-3 fatty acid metabolism simultaneously with symptom improvement [61]. Beyond the interesting findings of this study, some limitations should be considered, such as the use of patients who received medication in a study of biomarkers. Moreover, there was heterogeneity among the patients with BD regarding medication used at baseline, in follow-up, and in comorbid psychiatric disease [61]. This fact may limit the precision with which the findings of omega-3 fatty acid differences could be related to bipolar neuropathology. However, this study design maximized the generalizability of the findings to a real-world clinical population. Additionally, the above results at follow-up were only suggestive, as (i) treatment was not delivered as a controlled intervention, and (ii) less than 50% of the sample completed the follow-up [61].

Another study focused on the fact that affective disorders are usually associated with an increased risk of cardiovascular disease, which, at least partly, can appear to be independent of psychopharmacological treatments used to manage these disorders [62]. Reduced heart rate variability and a low omega-3 index have been shown to be associated with an increased risk of death after myocardial infarction. Therefore, Voggt et al. carried out a study to investigate heart rate variability and the omega-3 index in euthymic patients with BDs [62]. In fact, they assessed heart rate variability and the omega-3 index in 90 euthymic, mostly medicated patients with BDs (BD-I, BD-II), being free of significant medical comorbidity or stable psychotropic medication, and in 62 healthy individuals. The content of EPA + DHA in RBC membranes expressed as a percentage of total fatty acids was considered as the omega-3 index [62]. This study found that heart rate variability was considerably lower in patients with BDs compared to healthy individuals. In contrast, the omega-3 index did not differ considerably between the examined groups. Thus, the authors suggested that heart rate variability may provide a useful tool to study the impact of interventions aimed at reducing the increased risk of cardiovascular disease in euthymic patients with BDs. However, the difference in heart rate variability between cases and controls cannot be explained by a difference in the omega-3 index [62]. It should be noted that although RBCs appear to be an ideal biomarker for EPA + DHA intake, to date, the omega-3 index has not been measured using a standardized method.

In addition, McNamara et al. determined the composition of erythrocyte membrane fatty acids in first-episode bipolar manic or mixed (*n* = 40) and healthy (*n* = 40) individuals [63]. Erythrocyte fatty acid composition and clinical ratings were determined within a sub-group of bipolar individuals following 8-week (*n* = 19) or 52-week (*n* = 11) open-label treatment with lithium or quetiapine. At baseline, bipolar patients exhibited significantly lower erythrocyte DHA composition compared with healthy individuals (-23%). Both EPA and docosapentanoic acid were not different. Following an 8- or 52-week treatment with lithium or quetiapine, depressive and manic episodes decreased significantly [63]. On the other hand, erythrocyte fatty acids, including DHA, did not change. These data indicated that selective erythrocyte DHA deficits agreed with the initial onset of manic symptoms, and reductions in mood symptoms following treatment could not be mediated through alterations in fatty acids status [63]. Moreover, DHA levels did not differ between BD patients who smoked (*n* = 18) compared with those who did not smoke (*n* = 22). Although the gender interaction group was not significant for DHA, gender had a noteworthy main effect. All women (*n* = 40) exhibited higher DHA levels compared with all men (*n* = 40). Bipolar patients with (−26%) and without (−21%) attention deficit hyperactivity disorder exhibited similar DHA deficits compared with healthy individuals, and the two groups did not differ from each other [63].

One year later, the same research group performed another cross-sectional study to investigate long-chain omega-3 fatty acids status in youth with or at varying risk for developing mania and to assess its efficacy as a prodromic risk biomarker [64]. Erythrocyte fatty acid composition was determined in healthy adolescents (*n* = 28), asymptomatic adolescents with a biological parent with BD-I (*n* = 30; ‘high risk’, HR), adolescents with a biological parent with BD-I and MDD, or depressive disorder not otherwise specified (*n* = 36; ‘ultra-high risk’, UHR), and first-episode adolescent bipolar manic patients (*n* = 35, BP) [64]. Group differences were observed for both DHA and EPA. Compared with healthy individuals, erythrocyte EPA + DHA (omega-3 index) was notably lowered in BP (−24%) and UHR (−19%) groups and there was a trend in the HR group (−11%). In addition, compared with healthy individuals (61%), a greater percentage of HR (77%), UHR (80%), and BP (97%) patients exhibited EPA + DHA levels of ≤4.0% [64]. Among all participants (*n* = 130), EPA + DHA was inversely related to manic and depressive symptom severity. Thus, low EPA and DHA levels appeared to match with the initial onset of mania, and increasing risk for developing BD may be associated with graded erythrocyte EPA + DHA deficits. Together, these data suggested that low erythrocyte EPA + DHA status may represent a promising prodromic risk biomarker, justifying additional evaluation in future prospective studies [64]. A limitation of this study was the small number of individuals in each group, while the data obtained may not be representative of all individuals in each risk category. Moreover, this study did not administer a diet questionnaire to determine the contribution of habitual dietary patterns to the observed findings. Lastly, cross-sectional design prevented evaluation of causality, and therefore, prospective longitudinal studies were suggested to clarify the role of EPA + DHA status in bipolar risk progression [64].

In a double-blind random clinical trial, 100 patients with BD-I were randomly divided into two groups, i.e., control (*n* = 50) and experimental (*n* = 50) groups. In addition to the other standard treatments, 1000 mg of omega-3 fatty acids supplement was administered to the experimental group daily for 3 months, and placebo was given to the control group [65]. Before intervention, the mean severity of mania in the experimental group (23.50 ± 7.02) and control group (23.70 ± 8.09) was not significantly different. Notably, the difference after the intervention in the experimental group (10.64 ± 3.3) and control group (20.12 ± 6.78) was significant [65]. Since omega-3 fatty acid supplements were effective for the treatment of BD-I, it was suggested that omega-3 fatty acids supplements be used as adjuvant therapy along with other medication remedies. On the other hand, this study had some limitations as there was a dissimilarity of diet, and failure to measure blood levels of omega-3 fatty acids as well as inadequate evaluation by the Young Mania Rating Scale only at the beginning and end of the study and not on a weekly basis [65].

Two years later, another study aimed to characterize the cardiometabolic risk factors in a cohort of BD-I patients with limited exposure to psychotropic medications and to evaluate their associations with mood symptoms and omega-3 fatty acids blood levels [66]. Cardiometabolic risk assessments were compared in individuals with BD-I experiencing a first manic or mixed episode or an early depressive episode (*n* = 117) and healthy individuals (*n* = 56). Patients were medication-free at assessment and had no or limited exposure to mood stabilizers or antipsychotic medications prior to the admission. Following adjustment for demographic variables (i.e., age, gender, and parental education), significantly higher fasting triglyceride levels were observed in BD patients compared to healthy individuals. The omega-3 index was considerably lower in the bipolar group (3.4% vs. 3.9%). Within the bipolar group, no associations were found between the cardiometabolic parameters and the Clinical Global Impression-Severity Scale manic and depressive symptom ratings [66]. However, this study had certain limitations as it included a modest sample size. Moreover, there was a lack of sufficient data on inflammatory markers and a lack of sufficient metabolic data before the baseline evaluation to assess the duration of these metabolic abnormalities. The healthy individuals and patient groups were also not well matched in age, race, and education; however, this study controlled for these variables in its analyses [66]. The strengths of this study included the well-characterized cohort of recent-onset medication-free BD-I patients and the investigation of a range of different metabolic parameters. Collectively, the recent-onset medication-free BD was associated with higher triglyceride levels and lower omega-3 fatty acids levels. These findings are suggestive of early metabolic dysregulation that may progress over time and become exacerbated by exposure to psychotropic medications. Lower omega-3 fatty acid levels in BD patients may contribute to cardiometabolic risk and could, therefore, represent a therapeutic target for future prospective omega-3 fatty acids supplementation studies [66].

Inflammation and altered omega-3 fatty acid levels have been implicated in BD. In this aspect, Konga et al. performed a genome-wide association study to identify a locus in the fatty acid desaturase (FADS) gene cluster conferring susceptibility to BD [67]. This study examined omega-3 fatty acid levels in patients with BD in relation to proinflammatory cytokines, FADS genotype, and dietary habits. For this purpose, 83 patients with BD and 217 healthy individuals who underwent plasma omega-3 fatty acids measurement were enrolled. A subsample of 65 patients and 90 healthy individuals underwent plasma interleukin (IL)-6 and tumor necrosis factor alpha (TNFα) measurement, and three FADS single nucleotide polymorphisms (SNPs) were genotyped. Moreover, information on fish consumption was obtained from a self-reported diet history questionnaire [67]. Comparing omega-3 fatty acids levels between patients and healthy individuals, substantial differences were found for all seven PUFAs tested. In particular, the differences in EPA, DHA, γ-linolenic acid, AA, and EPA/AA ratio were highly significant. For the three omega-3 fatty acids, α-linolenic acid level was increased, while levels of EPA and DHA were decreased in BD patients compared to healthy individuals [67]. Plasma IL-6 and TNFα levels were both considerably increased in BD patients. Plasma EPA level was negatively associated with IL-6 and TNFα levels. The FADS genotype, which was associated with increased omega-6 fatty acids levels, was also associated with marked elevation in TNFα levels. EPA best correlated with oil-rich fish consumption in the combined cohort.

The above study also found that oil-rich fish consumption was associated with plasma DHA levels [67]. The frequency of oil-rich fish consumption, which correlated with plasma EPA level, was associated with high IL-6 levels. Taking together, these results provided strong evidence for altered plasma PUFAs and proinflammatory cytokine levels in patients with BD. Furthermore, FADS genotype and fish consumption may contribute not only to altered omega-3 fatty acids levels but also to inflammation in BD [Konga 2019]. However, this study had some limitations. Firstly, the severity of BD in the enrolled patients was moderately mild, and, therefore, further studies are necessary to examine whether severe bipolar cases could be related to more severe impairments in PUFAs, cytokines, and imbalances in dietary habits [67]. Secondly, the patients and healthy individuals differed in BMI, which may contribute to the observed difference in cytokine levels. Lastly, the cross-sectional design of this study made it difficult to determine whether the observed relationships were the causes or effects of the diseases. For this purpose, the authors suggested that prospective studies with dietary and genetic information or clinical trials of dietary intervention are needed to elucidate the possible causal role of PUFAs and cytokines in the pathogenesis of BD [67].

### 3.3. Clinical Studies in the Last Five Years: 2020–2024

BD is a severe mental disorder with a wide range of cognitive deficits, both in the euthymic and acute phases of the disease. In this respect, the aim of another study was to assess the impact of DHA supplementation on cognitive performances in euthymic BD patients [68]. This was an exploratory, single-center, double-blind, randomized controlled clinical trial evaluating 12 weeks of DHA supplementation (1250 mg daily) vs. a placebo (corn oil) in 31 euthymic BD patients compared to 15 healthy individuals on cognitive functions [68]. Overall, 31 BD patients (13 with omega-3 fatty acids and 18 with placebo) and 15 healthy individuals (7 with omega-3 and 8 with placebo) were enrolled. After 12 weeks of treatment, no significant group differences were observed in all neuropsychological tests between the four groups, except for the emotion inhibition test, where healthy individuals with DHA exhibited greater scores compared to either BD with DHA or BD with placebo. Moreover, significant differences were observed between healthy individuals and BD patients on Global Assessment of Functioning (GAF) scores and DHA dosages at baseline [68]. Specifically, it was found that BD patients with placebo had lower GAF scores compared to healthy individuals with or without omega-3 fatty acids treatment. Additionally, BD patients with omega-3 fatty acids showed lower GAF scores compared to healthy individuals and with placebo. Lastly, it was observed that healthy individuals with or without omega-3 fatty acids exhibited greater levels of DHA at baseline compared to BD with or without omega-3 fatty acids. Healthy individuals with omega-3 fatty acids exhibited lower mean age compared to BD patients with a placebo, while BD patients with a placebo had higher age compared to BD patients with omega-3 fatty acids. Although these results showed that DHA could be effective for ameliorating cognition in healthy subjects, future studies are still needed to clarify the impact of DHA on cognition in BD [68]. Moreover, this study had some limitations. Firstly, the sample size must be considered a meaningful limitation influencing the statistical power of study analyses. Secondly, the considerably higher age of BD patients compared with placebo could be a hypothetical reason for a greater cognitive dysfunction in patients with BD compared to healthy individuals [68]. Thirdly, the duration of this clinical trial could represent a potential bias since this time interval may not be sufficient to obtain substantial changes in brain fatty acids composition and, accordingly, to detect influences on cognition in patients with BD. Finally, the missing data from the last measurement of DHA plasma levels could represent a limitation for the complete interpretation of the results and the statistical analyses [68].

Moreover, a 12-week randomized, double-blind, parallel-group, placebo-controlled fixed-dose clinical trial was performed to assess the potential effects of fish oil monotherapy on depression and prefrontal neurochemistry in adolescents at high risk for BD-I [69]. All patients received three placebo or fish oil capsules daily. Each fish oil capsule contained 450 mg EPA, 40 mg docosapentaenoic acid (DPA), and 260 mg DHA for a total daily dose of 2130 mg EPA + DHA (1.7:1 EPA/DHA ratio) or 2250 mg omega-3 fatty acids (EPA + DPA + DHA) [69]. A total of 42 patients completed the 12-week clinical trial (placebo *n*  =  21 and fish oil *n*  =  21). Subjects randomized to fish oil, but not placebo, exhibited a significant baseline to endpoint increase in erythrocyte omega-3 fatty acids. EPA + DHA composition increased considerably from baseline in the fish oil group (+48%) but not in the placebo group (−9%). Fish oil produced a significantly greater decrease in Clinical Global Impression-Severity and Clinical Global Impression Improvement scores compared with placebo [69]. Overall, this study showed that fish oil monotherapy was not superior to placebo for reducing depressive symptoms in high-risk youth as assessed by the Childhood Depression Rating Scale-revised but was safe and well tolerated and superior to placebo on clinician ratings of global symptom improvement [69]. On the other hand, this study had some limitations. Firstly, the sample size was relatively small, and the data obtained may not be representative of all high-risk patients. Secondly, this study used a fixed dose of fish oil, and a different fixed-dose or a flexible dosing schedule may produce different results. Thirdly, the duration of fish oil supplementation was quite short (12 weeks), and greater reductions in depressive symptoms may require longer treatment [69]. On the other hand, study strengths included a well-characterized cohort of high-risk youth, the randomized, double-blind placebo-controlled study design, the closely matched treatment groups, and a relatively comprehensive panel of laboratory measures, including a membrane biomarker of omega-3 fatty acids status [69].

Another study assessed the efficacy of omega-3 fatty acids administration for prophylaxis in BD using a clinical trial design over 52 weeks [70]. Individuals with BD (*n* = 80) were randomized to receive a placebo (*n* = 40) or 1 g EPA plus 1 g DHA (*n* = 40) adjunctively for 52 weeks. The primary outcome measure comprised the number of mood episode relapses, including hospital admissions and medication changes experienced [70]. This study did not find significant differences in the number of mood episode relapses or the number of individuals requiring admission to the hospital or medication adjustment in the omega-3 fatty acids compared to the placebo group [70]. The time to relapse was not considerably different between groups. Notably, the change in Young Manic Rating Scale scores was considerably different between treatment groups over 12 months, with scores at 9 months and 12 months significantly lower compared to those at 3 months in the omega-3 fatty acids group and not in the placebo group [70]. However, changes in the Hamilton Depression Rating Scale, Global Clinical Impression, and Global Assessment of Functioning were not different between groups. Despite a minor reduction in hypomania scores in the omega-3 fatty acids group compared to placebo, this study found modest evidence of whether the supplementation of omega-3- fatty acids may exhibit prophylactic benefit in BD [70].

One year later, Saunders et al. investigated the preliminary efficacy of a high omega-3 fatty acids plus low omega-6 fatty acids dietary intervention in improving mood stability in BD when compared to dietary intervention with usual U.S. levels of omega-6 and omega-3 fatty acids intakes (control diet) [71]. This two-arm, parallel-group, randomized, modified double-blind, controlled 48-week clinical study of 12-week intensive diet intervention in patients with BD was conducted at a single suburban–rural site in the mid-Atlantic region. Participants with BD-I or BD-II with hypomanic or depressive symptoms were randomized and stratified on gender (*N* = 82). The intervention included the provision of group-specific study foods and dietary counseling [71]. Variability of mood symptoms was measured by a twice-daily, 12-week ecological momentary analysis paradigm, and group differences were analyzed using multilevel models. Ecological momentary analysis was designed to subjectively record mood ratings on a more frequent basis and in real time when individuals have been going about daily activities, allowing for a specific assessment of the variability of mood over time. This eliminated the need for the participant to recall and integrate mood shifts over time during reporting and provided a window into the daily experience of individuals with BD [71]. Circulating omega-3 fatty acids and omega-6 fatty acids were measured at baseline and after 4, 8, and 12 weeks of diet exposure. All 82 randomized participants were included in biochemical analyses. Seventy participants completed at least two ecological momentary analysis surveys and were included in primary ecological momentary analysis analyses. Variability in mood, energy, irritability, and pain, as measured using ecological momentary analysis, was reduced in the omega-3/omega-6 fatty acids group compared to the control diet group [71].

Concerning the above study, a multilevel analysis was also performed to identify differences in within-person variability between groups throughout the 12-week intensive intervention [71]. Controlling baseline symptoms, this study found that the omega-3/omega-6 fatty acids intervention group exhibited a considerably lower variability in mood, energy, irritability, and pain. Moreover, the omega-3/omega-6 intervention was successful in raising circulating DHA and EPA. The dietary intervention effect on target PUFAs significantly differed by the group over time [71]. On the other hand, no significant differences in mean ratings of mood symptoms or any other symptom measures were detected. The authors supported evidence that dietary intervention adjunctive to usual care showed preliminary efficacy in improving variability in mood symptoms in participants with BD [71]. A strength of this clinical trial was the use of two dietary-driven interventions delivered by dietitians with equal interaction in both groups, including dietary counseling and provision of whole foods. Additionally, the use of ecological momentary analysis was a strength of the clinical trial’s data collection mechanisms, allowing for the collection of rich and more frequently assessed data points to more accurately detect and analyze the sensitive variability of mood over a time course that was not frequently studied in treatment trials [71]. However, the sample size of this clinical trial was a limitation of its findings, and its main results need to be replicated in a larger clinical trial. Moreover, the sample was predominantly female, and the study was not powered to stratify analyses by gender. In addition, due to the complexity of the biological cascades, study results cannot prove whether changes in the omega-3 fatty acids or omega-6 fatty acids intake could be responsible for the improvement in mood through direct or indirect impacts [71].

Furthermore, a randomized, double-blind, controlled clinical trial of omega-3 fatty acids and inositol as monotherapies and in combination for the treatment of pediatric BD spectrum disorder was performed [72]. Participants were male and female children aged 5–12 years meeting DSM-IV diagnostic criteria for a BP spectrum disorder and displaying mixed, manic, or hypomanic symptoms without psychotic features at the time of evaluation. Participants concomitantly taking psychotropic medication were excluded from efficacy analyses. All participants received 975 mg EPA daily, with six soft gel capsules, which was 1650 mg combined EPA + DHA total daily dose, or six matched placebo capsules, daily [72]. Participants weighing ≥25 kg received 2000 mg (4 × 500 mg capsules) of inositol or placebo daily. Participants weighing <25 kg received 80 mg per kg rounded down to the nearest 500 mg capsule. This dose was maintained for the duration of the clinical trial and was allowed to be separated into two daily doses. Participants were randomized in a double-blind fashion into one of three treatment arms: inositol + placebo, omega-3 fatty acids + placebo, or inositol + omega-3 fatty acids [72]. Five participants were excluded from the efficacy analyses because they were taking adjunct mood-stabilizing medications throughout the clinical trial. Thus, the final groups for analysis included 17 participants in the omega-3 fatty acids group, 14 participants in the inositol group, and 16 in the combination group [72].

The above clinical study found that there were significant reductions in depressive and manic symptoms severity in the inositol and combination treatment groups and in Children’s Depression Rating Scale mean scores in the combination treatment group, with the largest changes seen in the combination group [72]. Those receiving the combination treatment showed the highest rates of antimanic and antidepressant response. All three treatment groups improved in symptoms of overall psychopathology and anxiety, with the combination group experiencing the greatest improvements in these domains [72]. The odds ratios for the combination group compared to the omega-3 fatty acids and inositol groups were clinically profound for 50% improvement on the Young Mania Rating Scale, normalization of the Young Mania Rating Scale (score <12) (vs. inositol group only), 50% improvement on the Hamilton Depression Rating Scale, 50% improvement on Children’s Depression Rating Scale (vs. omega-3 fatty acids group only), and Clinical Global Impression-I Mania, Clinical Global Impression-I MDD, and Clinical Global Impression-I Anxiety scores < 2 [72]. The strengths of this study included the double-blind design and the young participants, who were 5–12 years old. Additionally, no participants included in the efficacy analyses received concomitant antimanic or antidepressant medication beyond the study treatments. However, these results should be considered in the context of methodological limitations. The above findings may not be generalized to the general population, as the study sample consisted only of referred children. Additionally, since this study consisted largely of Caucasian children, its findings may not be generalizable to other ethnic groups. This clinical trial was also limited by a long recruitment period and a high dropout rate, attributable in part to the long study duration (12 weeks). On the other hand, the treatments were safe and easily available for over-the-counter purchase [72].

Although attention-deficit/hyperactivity disorder (ADHD) and a family history of BD-Ι increase the risk of developing BD, associated pathoetiological mechanisms remain poorly understood. One candidate risk factor is a neurodevelopmental deficiency in omega-3 fatty acids, including EPA and DHA. In this aspect, a cross-sectional study investigated erythrocyte EPA + DHA status in psychostimulant-free ADHD youth with (‘high-risk’, HR) and without (‘low-risk’, LR) a first-degree relative with BD, and healthy individuals [73]. A total of 123 (HR, *n* = 41; LR, *n* = 42; Healthy controls, *n* = 40) youth aged 10–18 years (mean age: 14.4 ± 2.5 years) were included in the analysis. Both HR and LR differed considerably from healthy individuals in terms of symptom ratings. HR had greater ADHD hyperactivity/impulsive symptom severity, manic symptom severity, and higher parent-reported ratings of internalization, externalization, and dysregulation compared with LR. Among all participants (*n* = 123), erythrocyte EPA + DHA levels were not substantially associated with BMI, age, or socioeconomic status, but they were positively associated with dietary EPA + DHA intake estimates [73]. A smaller percentage of HR exhibited an ‘omega-3 index’ (EPA + DHA) of ≥4.0 percent compared with LR, and there was a similar trend when compared with healthy individuals. ADHD youth with a BD family history exhibited erythrocyte EPA + DHA deficits and a more severe clinical profile, including greater manic and dysregulation symptoms, compared with ADHD youth without a BD family history [73]. The observation that ADHD youth with, but not without, a BD family history exhibited EPA + DHA deficits suggested a potential contribution of heritable etiological factors. For example, heritable polymorphisms in microsomal desaturase and elongase enzymes, which can mediate EPA and DHA biosynthesis from α-linolenic acid, have been characterized [74,75].

However, the above study had some limitations [73]. Firstly, the cross-sectional design prevented attributing causal relationships, and it was not known whether individuals in the different risk groups could develop BD in the future. Therefore, prospective longitudinal studies are warranted to evaluate whether erythrocyte EPA + DHA levels may affect BD development. Secondly, this study did not include a group of youth with syndromal BD to assess associations with symptom disease progression. However, as discussed in a previous study by the same research group, youth with syndromal BD, such patients may exhibit robust erythrocyte EPA + DHA deficits [64]. Study strengths included a well-characterized cohort of psychostimulant-free youth with ADHD with and without a BD family history, similar group demographics, assessment of relevant etiological variables, and a comprehensive assessment of symptom profiles [73].

As previously discussed, research suggests that a low omega-3 index may contribute to low heart rate variability and the increased risk of cardiovascular morbidity and mortality in BDs. In this aspect, Berger et al. explored the hypothesis of whether omega-3 fatty acids could considerably improve heart rate variability, measured as the standard deviation of the normal-to-normal interval (SDNN) [76]. In fact, they performed a randomized, double-blind controlled intervention clinical trial in euthymic BD patients with a low omega-3 index and reduced heart rate variability [76]. The omega index was estimated by EPA plus DHA expressed as a percentage of total identified fatty acids after response factor correction. This clinical trial was a single-center, 12-week parallel study comparison of omega-3 fatty acids vs. corn oil, in addition to the usual treatment. The primary endpoint of the clinical trial was a change in heart rate variability, as assessed by SDNN. Predefined secondary endpoints were a change in HRV, as assessed by a ratio of low frequency to high frequency (LF/HF), new episodes of bipolar depression, and mood rating scales [76]. Eligible patients were randomized to four capsules of EPAX 6015 TG per day (two in the morning, two in the evening), each containing 530 mg of EPA and 150 mg of DHA as triglycerides or four matching capsules containing corn oil as placebo, to receive with a meal to maximize bioavailability. The psychopathological state of the enrolled patients was determined using standardized rating scales: Young Mania Rating Scale, Hamilton Rating Scale for Depression, Montgomery–Åsberg Depression Rating Scale, Beck Depression Inventory, and Clinical Global Impressions Scale for Bipolar Illness [76].

In the above study, a total of 42 patients (omega-3: *n* = 23, corn oil: *n* = 19) finally completed the intervention after 12 weeks [76]. There was a considerable increase in the omega-3 index (value at endpoint minus value at baseline) in the omega-3 fatty acids group compared to the corn oil group. However, there was no significant difference in the change in the SDNN (value at endpoint minus value at baseline) between the treatment groups. In addition, non-association between changes in SDNN and changes in the omega-3 index was detected in the omega-3 fatty acids group or the corn oil group [76]. Similarly, no significant differences between corn oil and the omega-3 fatty acids group regarding the change in LF, HF, and LF/HF ratio were demonstrated. At baseline, there was a considerable difference in terms of the total score of the 21-item Hamilton Rating Scale for Depression scale. According to this scale, patients in the omega-3 fatty acids group were more depressed compared to those in the corn oil group, though they still did not meet the criteria for a depressive episode [76].

At the end of the above study, no significant differences between patients in the omega-3 fatty acids group and the corn oil group in none of the standardized rating scales were found [76]. In the intervention group, but not in the control group, the result of the regression model, with explaining variables EPA change, DHA change, and omega-3 index at baseline and age, gender, and diagnosis of BD as further co-variables in the model, indicated a positive association of the omega-3 index at baseline with an increase in SDNN during the study [76]. In addition, the change in DHA showed a positive association with concurrent change in SDNN in the study (i.e., increasing the concentration of DHA went along with increasing SDNN), while the change in EPA showed a negative association with SDNN (i.e., increasing concentration of EPA went along with decreasing SDNN) [76]. More to the point, the change in omega-3 index after 12 weeks (value at endpoint minus value at baseline) was −0.4 ± 1.06% in the corn oil group, compared to 5.0 ± 1.94% in the omega-3 fatty acids group. Taken together, in this randomized, controlled intervention clinical trial in euthymic bipolar patients with a low omega-3 index and reduced heart rate variability, no significant effects of omega-3 fatty acids on SDNN or frequency-domain measures HF, LF and LF/HF ratio were detected. Possible reasons included, among others, the effect of psychotropic medication present in this clinical trial and/or the genetics of BD itself [76].

A Mendelian randomization study based on genome-wide association study (GWAS) data was performed to explore the causal association between omega-3 fatty acids and omega-6 fatty acids and BD [77]. The effects of omega-3 fatty acids and omega-6 fatty acids on BD endpoints were assessed in up to 34,950 individuals of European ancestry using the largest PUFA GWAS to date, an exposed sample containing 114,999 individuals [77]. This study showed that increased omega-3 fatty acid levels were negatively and causally associated with the risk of BD. This study has the advantage of applying a Mendelian randomization analysis, which can prevent reverse causation caused by the confounding factors inherent in traditional observational studies. Thus, its results should be considered robust after testing for heterogeneity and pleiotropy [77]. However, this study still has some limitations. Firstly, this study used publicly available data from the UK Biobank and EBI databases, and therefore, the study population was mainly from European countries. Hence, although bias caused by different populations was avoided, the generalizability of its research findings seems uncertain [77]. Secondly, the basic information of the study cohort was not directly available, and it was unable to obtain the socioeconomic status, BMI levels, and dietary status of individuals to adjust for potential confounding factors. Thirdly, due to limitations of the available public databases, this study only used the total levels of fatty acids and did not perform a more detailed analysis, including the analysis of DHA, EPA, and other omega-3 fatty acids [77].

Inflammation accelerates the progression of BD, and supplementation of anti-inflammatory supplements in adjuvant with medications may alleviate disorder signs. In this aspect, a randomized double-blind controlled clinical trial aimed to investigate the effects of omega-3 fatty acids supplementation on the serum concentrations of pro-inflammatory cytokines and depression status in patients with BD [78]. Patients with BD (*n* = 60) were split into two groups: omega-3 fatty acids supplement group (*n* = 30, 15 men and 15 women) and placebo one using a permuted block stratified randomization. The patients in the omega-3 fatty acids group received 2 g of omega-3 fatty acids daily for 2 months, while the patients in the placebo group received 2 g soft gels daily in the same form. In fact, the patients in the omega-3 fatty acids group daily consumed two capsules of 1000 mg of omega-3 fatty acids (180 mg EPA, 120 mg DHA) for 2 months after meals with a sufficient amount of water. Depression score and the serum concentrations of tumor necrosis factor-α (TNF-α), interleukin-6 (IL-6), and high-sensitivity C-reactive protein (hs-CRP) were assessed before and after the study [78]. Depressive symptoms and the serum concentrations of TNF-α, IL-6, and hs-CRP were decreased after intervention in the omega-3 fatty acids group compared with the placebo group. The serum concentrations of IL-6, TNF-α, and hs-CRP were also decreased by 50.00%, 32.00%, and 43.00%, respectively, after the intervention compared with before intervention in the case group. The results also showed a positive correlation between the serum concentrations of TNF-α, IL-6, and hs-CRP with depression scores before and after intervention in both groups. However, this study had some limitations, such as assessing cytokines in serum may not reflect their concentrations in the brain [78]. Although studies have reported that changes in peripheral levels may partly reflect the changes in brain cytokine secretion, they did not find any definitive conclusion on this matter [78]. This study investigated only three inflammatory factors, which was a limitation of this study, and assessing other factors could be a better idea for future studies. The lack of a follow-up period was another limitation of this study. On the other hand, the strength point of this study was that it was novel and could open new doors for future researchers and help to manage the treatment of BD [78].

Antioxidants may prevent the progression of neuropsychiatric disorders, such as BD. Omega-3 fatty acid supplementation can contribute to the prevention of lipid peroxidation, improving antioxidant status. BD imbalances, oxidant–antioxidant balance, and omega-3 fatty acids supplementation may improve the balance in patients with BD. In addition, examining the intake and metabolism of omega-3 fatty acids supplementation has provided clues that may be crucial for treating BD. In this aspect, Gholipour et al. performed a randomized, double-blind, controlled clinical trial to investigate the effect of omega-3 fatty acids supplementation on serum levels of antioxidant status in 56 Iranian patients with BD [79]. In this study, 28 patients with BD and a male/female ratio equal to 1:1 received an omega-3 fatty acid supplement (2 g/daily), while the other 28 patients received edible paraffin oil (2 g/daily) for 60 days. The results showed that omega-3 fatty acids supplementation increased the activities of superoxide dismutase (21%) and catalase (27%) in post-intervention compared to pre-intervention [79]. The results also showed that omega-3 fatty acid supplementation increased the activities of superoxide dismutase and catalase compared to the control group in post-intervention. However, omega-3 fatty acids supplementation did not have significant effects on the serum concentration of total antioxidant capacity compared to the pre-intervention and control group, which may be ascribed to different efficiency and mechanisms of omega-3 fatty acids on various antioxidant parameters. Taken together, this study revealed that omega-3 fatty acids supplementation increased the activities of superoxide dismutase and catalase and may decrease the progression of disease via increasing antioxidant status [79].

Recently, a 6-month pilot randomized controlled clinical trial aimed to assess the prophylactic effects and tolerability of high-dose omega-3 fatty acids in the prevention of relapse of bipolar depression [80]. In fact, 31 stable BD patients were randomized to receive omega-3 fatty acids (*n* = 16) or placebo (*n* = 15) for 6 months, and intergroup differences in the incidence of the recurrence of bipolar depression were assessed. Eligible patients were randomized to receive fish oil capsules (420 mg EPA, 220 mg DHA, 0.2 mg tertiary-butylhydroquinone, and 2.0 mg vitamin E) or a placebo (soybean oil) [80]. The patients took four capsules daily for 6 months, totaling 1680 mg EPA and 880 mg DHA per day. This study showed that patients in the omega-3 fatty acids group had a significantly lower incidence of the recurrence of bipolar depression compared to those in the placebo group. Similarly, a lower Hamilton Rating Scale for Depression score was noted in the omega-3 fatty acids group at the 2nd, 3rd, 4th, 5th, and 6th months [80]. Furthermore, repeated measures ANOVA indicated a significant effect of time and group on the Hamilton Rating Scale for Depression scores. Omega-3 fatty acids were associated with increased levels of glutamate oxaloacetate transaminase, glutamate pyruvate transaminase, and blood urea nitrogen (BUN) at the end point [80]. The patients involved in this study were normal for routine biochemical parameters and had no psychiatric or somatic comorbidities, which could potentially enhance the prophylactic effects of omega-3 fatty acids on bipolar depression. However, the levels of these biomarkers were within the normal physiological thresholds and did not pose any risk of adverse effects, consistent with the safety and tolerability profiles of omega-3 fatty acids [80]. Of note, this study used an EPA-predominant formulation, which was shown to have more antidepressant efficacy [80].

Another recent study utilized MR to conduct a thorough investigation into the relationship between 28 prevalent dietary habits and BD [81]. An analysis was conducted using publicly available genome-wide association study data from the UK Biobank dataset. UK Biobank dataset is a large-scale prospective study involving approximately 500,000 participants aged between 38 and 73, providing genetic and phenotypic information [81]. Intake of non-oily fish and sponge pudding both had a positive association with BD [81]. Oily fish, dried fruit, apples, salt, and cooked vegetable intake also appeared potentially risky for BD, although the possibility of false positives cannot be ruled out. Sensitivity analysis further confirmed the robustness of these findings. This research provided evidence of a relationship between various dietary habits and BD. It underscored the need for careful dietary management and balance to reduce the risk of BD, suggesting caution with dietary preferences for fish and sponge pudding [81]. Despite this study being one of the most comprehensive MR studies to assess the role of diet on BD, it involved some limitations. Firstly, its methodology mainly focused on individuals of European descent; therefore, it may limit its generalizability. Secondly, while it deliberately focused on examining the relationships between specific dietary exposures and outcomes based on certain literature, this approach to selecting diets was not entirely systematic and may lack comprehensiveness [81]. Thirdly, the original data utilized in the MR analysis were not segregated by gender, lacked standardized diagnostic criteria, and did not account for comorbidities, which could introduce gender-specific biases and variabilities into the analysis. Lastly, the complexity and interplay of dietary components may suggest that focusing solely on individual nutrients or food groups may be flawed. Nutrient interactions and antagonisms of varying degrees within various diets could make the relationship between dietary habits and mental health even more complex [81].

**Table 1:** Clinical human studies evaluating the role of omega-3 fatty acids in BD symptoms.

Authors/Ref.	Type of Study and Population	Existing Conventional Therapy	Omega-3 Treatment and Period	Main Results
Stroll et al., 1999 [32]	Double-blind, placebo-controlled RCT, *n* = 30 BD patients.	Lithium medication treatment.	6.2 g of EPA and 3.4 g of DHA daily for 4 months.	The omega-3 fatty acids patient group exhibited a substantially longer period of remission compared to the placebo group. For nearly every other secondary outcome measure (YMRS, HRSD, CGI, GAS), the omega-3 fatty acid group performed better than the placebo group.
Chiu et al., 2003 [33]	Double-blind, placebo-controlled RCT, *n* = 20 bipolar manic patients and *n* = 20 healthy controls	No medication treatment.	6 g EPA daily for 4 months.	Arachidonic acid and DHA compositions in erythrocyte membranes were substantially lowered in BD patients compared to normal controls.
Noaghiul et al., 2003 [34]	Cross-national study of 12 countries assessing lifetime prevalence rate for bipolar spectrum disorder.	Not applicable.	Seafood consumption (3 g EPA plus DHA) daily.	Greater seafood consumption predicted lower lifetime frequency rates of BD-I, BD-II, and bipolar spectrum disorder.
Hirashima et al., 2004 [35]	Double-blind, placebo-controlled RCT, *n* = 12 BD female patients received omega-3 fatty acids. BD female patients (*n* = 9) and a group without BD (*n* = 12) did not receive omega-3 fatty acids.	Bipolar subjects received lithium (*N* = 7), valproic acid (*N* = 10), other anticonvulsants (*N* = 2), or no medications (*N* = 2) before their initial MRI scans.	High dose of 5.0–5.2 g/day of EPA, 3.0–3.4 g/day of DHA, and 1.7 g/day of other omega-3 fatty acids (*N* = 6) or low dose of a total of 1.3 g/day of EPA acid and 0.7 g/day of DHA (*N* = 6) for 4 weeks.	Both the omega-3 fatty acids high-dose and omega-3 fatty acids low-dose groups showed substantial reductions in percentage change in MRI T2 median times compared to the comparison group. No significant changes in HDRS or YMRS scores in the three groups from the baseline to the 4th week.
Osher et al., 2005 [36]	Open-label add-on CT, *n* = 12 BD-I outpatients.	No medication treatment.	1.5 to 2 g/day of EPA for up to 6 months.	Eight of the 10 patients who completed at least one month of follow-up accomplished a 50% or greater reduction in HDRS scores within one month. No patients developed hypomania or manic symptoms.
Sagduyu et al., 2005 [37]	Open-ended, add-on CT, *n* = 37 BD patients.	Not available received medications treatment.	The mean starting dose was 1824.32 mg EPA + DHA, and the mean for the last maintenance dose was considerably higher at 2878.38 mg. The mean duration was 439.62 days.	Omega-3 fatty acids added to the existing treatment helped with the irritability component of a significant percentage of BD patients with a persistent sign of irritability. The optimum effective dose for irritability was 1–2 gr of EPA plus DHA per day low dose (1 to 2 g per day).
Keck et al., 2006 [38]	Double-blind, placebo-controlled RCT, *n* = 116 BD patients.	Patients received at least one mood-stabilizing medication (not available).	Ethyl-EPA 6 g/day for 4 months.	There were no significant differences in any outcome measure (depressive or manic symptoms) between the ethyl-EPA and placebo groups.
Frangou et al., 2006 [39]	Double-blind, placebo-controlled RCT, *n* = 75 BD patients.	Not available received medications treatment.	1 g/day or 2 g/day of ethyl-EPA for 12 weeks.	There was no apparent benefit of 2 g over 1 g ethyl-EPA daily. Significant improvement was noted with ethyl-EPA treatment compared with placebo in the HDRS and the CGI scores.
Marangell et al., 2006 [40]	Double-blind, placebo-controlled RCT, *n* = 10 BD female patients.	No medication treatment.	2000 mg/day DHA for one year.	The data were not considered sufficient to support a recommendation of monotherapy treatment as a substitute for standard pharmacologic treatments.
Fragou et al., 2007 [41]	Double-blind, placebo-controlled RCT, *n* = 14 BD female patients.	Lithium medication treatment.	2 g of ethyl-EPA per day for 12 weeks.	A significant rise in N-acetylaspartate levels (a putative marker of neuronal integrity) was observed in the ethyl-EPA treatment group compared with the placebo group.
Wozniak et al., 2007 [42]	Prospective open-label CT, *n* = 20 children and adolescents with BD.	No medication treatment.	1290–4300 mg combined EPA and DHA for 8 weeks.	Greater clinical improvement was observed based on YMRS and CDRS scores.
Sublette et al., 2007 [44]	Observational, parallel-group, cross-sectional CT, *n* = 10 BD patients, *n* = 10 healthy volunteers.	No medication treatment.	No omega-3 fatty acids or other treatment.	No between-group differences were found in levels of individuals or total fatty acids (EPA and DHA), or of prostaglandin E2. Manic symptom severity was negatively associated with levels of free arachidonic acid and free EPA and positively with the free arachidonic acid/EPA ratio.
Clayton et al., 2008 [45]	Observational, parallel-group, cross-sectional CT, *n* = 15 BD children and adolescents with juvenile BD, *n* = 15 healthy volunteers.	Mood-stabilizing medication treatment.	No omega-3 fatty acids or other treatment.	RBC membrane concentrations of EPA and DHA were not significantly lower in JBD participants compared with healthy controls. RBC DHA was negatively related to clinician ratings of depression. Plasma EPA% was positively related to clinician ratings of mania.
McNamara et al., 2008 [46]	Postmortem orbitofrontal cortex study of patients with BD (*n* = 18) and normal controls (*n* = 19).	Mood-stabilizing medication (lithium, valproic acid, and/or carbamazepine) medication treatment.	No omega-3 fatty acids or other treatment.	DHA (−24%), arachidonic acid (−14%), and stearic acid (−4.5%) compositions were significantly lower in orbitofrontal cortex BD patients. BD patients who died by suicide exhibited selective deficits in orbitofrontal cortex DHA composition.
Clayton et al., 2009 [47]	Open-label CT, *n* = 15 children and adolescents with juvenile BD.	Mood-stabilizing medication treatment.	360 mg/day EPA and 1560 mg/day DHA for 6 weeks.	RBC EPA and DHA were considerably higher after intervention. Clinician ratings of mania and depression were substantially lower, and global functioning was considerably higher after intervention.
McNamara et al., 2010 [48]	Observational, parallel-group, cross-sectional CT, *n* = 20 BD patients, *n* = 20, MDD patients, *n* = 20, Healthy controls.	No medication treatment.	No omega-3 fatty acids or other treatment.	Both BD (−32%) and MDD (−20%) patients exhibited significantly lower erythrocyte DHA composition relative to healthy controls. Erythrocyte DHA composition was not substantially associated with depression or mania symptom severity scores.
Gracious et al., 2010 [56]	Placebo-controlled RCT, *n* = 51 children and adolescents with BD, manic, hypomanic, mixed, or depressed behavior.	Lithium medication treatment.	Flax oil capsules containing 550 mg α-linolenic acid per 1 g or an olive oil placebo adjunctively or as monotherapy. Doses were titrated to 12 capsules per day as tolerated over 16 weeks (up to 6.6 g of daily α-linolenic acid)	There were no significant differences in primary outcome measures when compared by treatment assignment (YMRS, CDRS, and CGI scores). Clinician-rated Global Symptom Severity was negatively associated with final serum omega-3 fatty acid compositions: % α-linolenic acid, % EPA, and positively associated with final DHA.
Murphy et al., 2012 [57]	Double-blind, placebo-controlled add-on CT, *n* = 51 BD patients.	Mood-stabilizing anticonvulsants, lithium, antipsychotic drugs, antidepressant drugs, and antianxiety agents medication treatment.	(1) oral cytidine + omega-3 fatty acids, (2) placebo + omega-3 fatty acids, and (3) placebo + placebo control Fish oil was given as 4 capsules twice a day (approximately 3 g of EPA/daily), and cytidine was administered as 4 capsules twice a day (2 g cytidine/daily) for 4 months.	Clinical measures improved in all treatment groups, and there were no significant differences between groups, including changes in the probability of symptoms of depression or mania, changes in positive ratings of depression or mania, or changes in GAF scores.
Pomponi et al., 2013 [58]	Observational, parallel-group, cross-sectional CT, *n* = 42 BD patients (Depressed, *n* = 21, Manic, *n* = 9, Euthymic, *n* = 12, Healthy controls, *n* = 57).	Mood stabilizers, antimanic drugs, and antidepressant medication treatment.	No omega-3 fatty acids or other treatment.	Plasma DHA levels were substantially decreased in BD patients. Compared with controls, patients exhibited greater plasma levels of all other fatty acids, including arachidonic acid, alpha-linolenic acid.
Wozniak et al., 2015 [59]	Double-blind, placebo-controlled RCT, *n* = 24 children with BD spectrum disorders.	Children weighing ≥25 kg were dosed at 2000 mg (4 × 500 mg capsules) of inositol or placebo daily, and those weighing <25 kg were dosed at 80 mg per kg.	All participants were dosed 975 mg EPA daily, with 6 soft gel capsules, which were 1650 mg combined EPA + DHA total daily dose, or 6 matched placebo capsules daily for 12 weeks.	The combined treatment of omega-3 fatty acids plus inositol reduced symptoms of mania and depression in prepubertal children with mild to moderate BD spectrum disorders.
Fristad et al., 2015 [60]	Double-blind, placebo-controlled RCT, *n* = 23 youth with BD-NOS or cyclothymic disorder.	No medication treatment except for stable medication for attention-deficit/hyperactivity disorder and sleep aids.	Omega-3 fatty acids groups received two 500 mg omega-3 fatty acids capsules (350 mg EPA, 50 mg DHA; 100 mg other omega-3) twice daily for a total daily dose of 2000 mg of omega-3 fatty acids (1400 mg EPA, 200 mg DHA; 400 mg other) for 12 weeks.	Significant improvement in depressive symptoms (KDRS) for combined treatment compared to placebo and active monitoring. Across groups, manic symptoms improved over time without significant treatment effects.
Saunders et al., 2015 [61]	Observational, parallel-group, cross-sectional CT, *n* = 27 BD patients and *n* = 31 healthy controls.	Antidepressant, antipsychotic, or mood-stabilizing medication treatment.	No omega-3 fatty acids or other treatment.	Unesterified EPA was lower in BD than healthy controls, with a large effect size. Exploratory comparison revealed lower unesterified/esterified EPA in BD compared to healthy controls. Mania severity and suicidality were positively associated with unesterified/esterified EPA ratio.
Voggt et al., 2015 [62]	Observational, parallel-group CT, *n* = 90 BD patients and *n* = 62 healthy volunteers.	No medication treatment.	No omega-3 fatty acids or other treatment.	The omega-3 index (EPA + DHA in RBC membranes expressed as a percentage of total fatty acids) did not differ significantly between the groups.
McNamara et al., 2015 [63]	Open-label, parallel-group, cross-sectional CT, first-episode bipolar manic or mixed (*n* = 40) and healthy (*n* = 40) individuals.	Lithium or quetiapine medication treatment.	Bipolar individuals followed up for 8 weeks (*n* = 19) or 52 weeks (*n* = 11) during open-label treatment with lithium or quetiapine.	At baseline, bipolar patients exhibited significantly lower erythrocyte DHA composition compared with healthy individuals. Treatment with lithium or quetiapine reduced depressive and manic symptoms. Erythrocyte fatty acids, including DHA, did not change during the treatment.
McNamara et al., 2016 [64]	Open-label, parallel-group, cross-sectional CT, asymptomatic adolescents with a biological parent with BD-I (*n* = 30; ‘high risk’, HR), adolescents with a biological parent with BD-I and major depressive disorder, or depressive disorder not otherwise specified (*n* = 36; ‘ultra-high risk’, UHR), and first-episode adolescent bipolar manic patients (*n* = 35, BP) and *n* = 28 healthy adolescents.	No medication treatment.	No omega-3 fatty acids or other treatment.	Compared with healthy controls, erythrocyte EPA + DHA (omega-3 index) was notably lowered in BP (−24%) and UHR (−19%) groups, and there was a trend in the HR group (−11%). Compared with healthy controls (61%), a greater percentage of HR (77%), UHR (80%), and BP (97%) patients exhibited EPA + DHA levels of ≤4.0%. Among all participants (*n* = 130), EPA + DHA was inversely related to manic and depressive symptom severity.
Shakeri et al., 2016 [65]	Double-blind, placebo-controlled RCT, *n* = 50 healthy controls, and *n* = 50 patients with BD-I.	Lithium and olanzapine medication treatment.	1000 mg of omega-3 fatty acids supplement was given to the experimental group on a daily basis for 3 months.	Before intervention, the mean severity of mania in the experimental group (23.50 ± 7.02) and control group (23.70 ± 8.09) was not significant using YMRS. The difference after the intervention concerning the mean severity of mania in the patients’ group (10.64 ± 3.3) and healthy control group (20.12 ± 6.78) was significant using YMRS.
Wulsin et al., 2018 [66]	Open-label, parallel-group, cross-sectional CT, *n* = 117 patients with BD-I and *n* = 56 healthy individuals.	No medication treatment.	No omega-3 fatty acids or other treatment.	The omega-3 index was significantly lower in the BD-I patients compared to healthy individuals (3.4% vs. 3.9%).
Koga et al., 2019 [67]	Open-label, parallel-group, cross-sectional CT, *n* = 83 patients with BD and *n* = 217 healthy individuals.	Not available received medications treatment.	No omega-3 fatty acids or other treatment.	Plasma EPA and DHA levels were decreased in patients compared to healthy individuals. Plasma IL-6 and TNFα levels were both considerably increased in BD patients. Plasma EPA level was negatively associated with IL-6 and TNFα levels.
Ciappolino et al., 2020 [68]	Double-blind, placebo-controlled RCT, *n* = 31 euthymic BD patients, and *n* = 15 healthy individuals.	Not available received medications treatment.	DHA supplementation (1250 mg daily) for 12 weeks	No significant group differences were observed in all neuropsychological tests between the four groups, except for the emotion inhibition test, where healthy controls with DHA exhibited greater scores compared to either BD with DHA or BD with placebo.
McNamara et al., 2020 [69]	Double-blind, placebo-controlled RCT, placebo *n* = 21 and fish oil *n* = 21 adolescents at high risk for BD-I.	No medication treatment.	A total daily dose of 2130 mg EPA + DHA (1.7:1 EPA/DHA ratio) or 2250 mg omega-3 PUFAs (EPA + DPA + DHA) for 12 weeks.	EPA + DHA composition increased significantly from baseline in the fish oil group (+48%) but not in the placebo group (−9%). Fish oil produced a significantly greater decrease in Clinical Global Impression-Severity and Clinical Global Impression Improvement scores compared with placebo.
McPhilemy et al., 2021 [70]	Double-blind, placebo-controlled, parallel-group RCT, *n* = 80 patients with BD-I.	Antidepressant, antipsychotic, or mood-stabilizing medication treatment.	1 g EPA plus 1 g DHA (*n* = 40) adjunctively for 52 weeks.	No significant differences in the number of mood episode relapses or the number of individuals requiring admission to the hospital or medication adjustment in the omega-3 fatty acids compared to the placebo group. Change in Young Manic Rating Scale was considerably different between treatment groups over 12 months, with scores at 9 months and 12 months significantly lower compared to those at 3 months in the omega-3 group and not in the placebo group.
Saunders et al., 2022 [71]	Double-blind, placebo-controlled 2-arm, parallel-group RCT, *n* = 82 patients with BD-I or BD-II.	Not available received medications treatment.	Omega-3 fatty acids EPA + DHA (1500 mg per day) plus low omega-6 linoleic acid diet or a control diet standardized to the usual American distribution of omega-3 fatty acids EPA + DHA (150 mg per day) and omega-6 linoleic acid for 12 weeks.	Variability in mood, energy, irritability, and pain, as measured using ecological momentary analysis, was reduced in the omega-3/omega-6 group compared to the control diet group. The omega-3/omega-6 intervention group exhibited a considerably lower variability in mood, energy, irritability, and pain. The omega-3/omega-6 intervention was successful in raising circulating DHA and EPA. Dietary intervention adjunctive to usual care showed preliminary efficacy in improving variability in mood symptoms in participants with BD.
Wozniak et al., 2022 [72]	Double-blind, placebo-controlled, parallel-group RCT, *n* = 27 children ages 5–12 with a bipolar spectrum disorder.	No medication treatment.	All participants were dosed at 975 mg EPA daily, with 6 of the soft gel capsules, which was 1650 mg combined EPA + DHA total daily dose, or 6 matched placebo capsules, daily for 12 weeks. Participants weighing ≥25 kg were dosed at 2000 mg (4500 mg capsules) of inositol or placebo daily. Participants weighing <25 kg were dosed at 80 mg per kg rounded down to the nearest 500 mg capsule for 12 weeks.	There were significant reductions in YMRS and HDRS mean scores in the inositol and combination treatment groups and in CDRS mean scores in the combination treatment group, with the largest changes seen in the combination group. Those receiving the combination treatment had the highest rates of antimanic and antidepressant response.
McNamara et al., 2022 [73]	Open-label, parallel-group, cross-sectional CT, ADHD youth with high-risk (*n* = 41), and without (‘low-risk’, *n* = 42) a first-degree relative with BD, and healthy controls (*n* = 40).	No medication treatment.	No omega-3 fatty acids or other treatment.	Among all participants (*n* = 123), erythrocyte EPA + DHA was not substantially associated with BMI, age, or socioeconomic status but was positively associated with dietary EPA + DHA intake estimates. A smaller percentage of high-risk youth exhibited an ‘omega-3 index’ (EPA + DHA) of ≥4.0 percent compared with low-risk youth. ADHD youth with a BD family history exhibited erythrocyte EPA + DHA deficits and a more severe clinical profile, including greater manic and dysregulation symptoms, compared with ADHD youth and those without a BD family history.
Berger et al., 2022 [76]	Double-blind, placebo-controlled, parallel-group RCT, omega-3 fatty acids: *n* = 23 intervention, corn oil: *n* = 19 placebo group of patients with BD-I or BD-II.	Multiple psychotropic medications received.	4 capsules EPAX 6015 TG per day (2 in the morning, 2 in the evening), each containing 530 mg of EPA and 150 mg of DHA or 4 matching capsules containing corn oil as a placebo for 12 weeks.	Significant increase in the omega-3 index (value at endpoint minus value at baseline) in the omega-3 group compared to the corn oil group. Patients in the omega-3 fatty acids group were more depressed than those in the corn oil group, though still did not meet the criteria for a depressive episode. The change in DHA showed a positive association with the concurrent change in SDNN in the study (i.e., increasing the concentration of DHA went along with increasing SDNN), while the change in EPA showed a negative association with SDNN.
Zhang et al., 2023 [77]	2-sample bidirectional mendelian randomization study, UK Biobank and EBI databases, *n* = 7647 BD patients and *n* = 20,998 healthy controls	Lithium medications treatment.	No omega-3 fatty acids or other treatment.	Increased omega-3 fatty acids were negatively and causally associated with the risk of BD.
Eshlahi et al., 2023 [78]	Double-blind, placebo-controlled, parallel-group RCT. Patients with BD (*n* = 60) were grouped into two groups: the omega-3 fatty acid supplement group (*n* = 30) and the placebo one (*n* = 30).	Not available received medications treatment.	The patients in the omega-3 fatty acid group consumed 2 capsules of 1000 mg of omega-3 fatty acids daily (180 mg EPA, 120 mg DHA) for 2 months.	Depression score and the serum concentrations of TNF-α, IL-6, and hs-CRP were decreased after intervention in the omega-3 fatty acids group compared with the placebo group.
Gholipour et al., 2024 [79]	Double-blind, placebo-controlled, parallel-group RCT, *n* = 56 BD patients.	Not available received medications treatment.	2 g of omega-3 fatty acids in gel form in the intervention group and 2 g of paraffin in the placebo group for 2 months.	Omega-3 fatty acids supplementation increased the activities of superoxide dismutase and catalase in post-intervention compared to pre-intervention. Omega-3 fatty acid supplementation increased the activities of superoxide dismutase and catalase compared to the control group post-intervention.
Zailani et al., 2024 [80]	Parallel-group, double-blind CTs, *n* = 31 BD patients.	Lithium or valproate medication treatment.	1680 mg EPA and 880 mg DHA per day for 6 months.	The omega-3 fatty acids group had a significantly lower incidence of the recurrence of bipolar depression compared to those in the placebo group. A lower HDRS score was noted in the omega-3 fatty acids group at the 2nd, 3rd, 4th, 5th, and 6th months. Omega-3 fatty acids were associated with increased levels of glutamate oxaloacetate transaminase, glutamate pyruvate transaminase, and blood urea nitrogen.
Li et al., 2024 [81]	Mendelian RCT, UK Biobank, *n* = 41,917 BD cases and *n* = 371,549 controls of European ancestry.	Not available received medications treatment.	No omega-3 fatty acids or other treatment.	Intake of non-oily fish and sponge pudding had a positive association with BD.

RCT: randomized clinical trial; BD: bipolar disorder; EPA: eicosapentaenoic acid; DHA: docosahexaenoic acid; YMRS: Young Mania Rating Scale; HDRS: Hamilton Depression Rating Scale; CGI: Clinical Global Impression; GAS: Global Assessment Scale; MRI: magnetic resonance imaging; CDRS: Child Depression Rating Scales; PUFAs: polyunsaturated fatty acids; RBC: red blood cell; MDD: major depression disease; GAF: Global Assessment of Functioning; BD-NOS: bipolar disorder not otherwise specified, IL-6: interleukin-6, TNFα: tumor necrosis factor α, DPA: docosapentaenoic acid, ADHD: attention-deficit/hyperactivity disorder, SDNN: standard deviation of the normal to normal interval.

There are some meta-analysis studies concerning the potential effect of omega-3 fatty acids in BD patients. In this aspect, Sarris et al. performed a meta-analysis including randomized controlled studies with a duration of 4 weeks or longer, with a sample size > 10, written in English, and using omega-3 fatty acids for diagnosed bipolar depression or mania [82]. No criteria were set for age, gender, or ethnicity. The findings of five pooled datasets (*n* = 291) on the outcome of bipolar depression revealed a significant effect in favor of omega-3 fatty acids. On the outcome of mania, five pooled datasets (*n* = 291) revealed a nonsignificant effect in favor of omega-3 fatty acids [82]. Minor heterogeneity between studies on the outcome of bipolar depression was found, which was not present in the outcome of bipolar mania. Funnel plot symmetry suggested no significant likelihood of publication bias. Meta-regression analysis between sample size and effect size, however, revealed that studies with smaller sample sizes had larger effect sizes. These meta-analytic findings provided evidence that bipolar depressive symptoms may be improved by adjunctive use of omega-3 fatty acids. The evidence, however, did not support its adjunctive use in attenuating mania [82].

A more recent meta-analysis of eight double-blind, randomized, placebo-controlled clinical trials of omega-3 fatty acids (DHA, EPA, or both DHA and EPA treatment (*n* = 338)) for BD was reported [83]. This meta-analysis detected that omega-3 fatty acids intake was superior to placebo in the improvement of residual depressive symptoms and all-cause discontinuation. Because two studies contributed to the selection or attrition biases, a sensitivity analysis was conducted, excluding these studies from the primary outcome [83]. This sensitivity analysis revealed the superiority of omega-3 fatty acids over placebo, but considerable heterogeneity disappeared, suggesting that these studies’ data may cause considerable heterogeneity. Thus, this meta-analysis suggested that adjunctive therapy of omega-3 fatty acids may positively contribute to the treatment of residual depressive symptoms in BD. However, because it may include a small-study effect, larger double-blind, randomized, placebo-controlled clinical trials should be conducted to investigate whether these results can be replicated [83].

## 4. Discussion

### 4.1. Current Clinical Evidence

We comprehensively searched several accurate international literature databases, retrieving a rather small number of in vitro and in vivo animal studies as well as clinical human studies concerning the potential effect of omega-3 fatty treatment in BD. After applying the described exclusion criteria, we identified 40 clinical human studies highlighting the role of omega-3 acids in BD. The first clinical study was published 26 years ago by Stroll et al. in 1999 [32]. When searching for other natural compounds, the number of clinical studies was much less and very scarce. Thus, it appears that among the natural compounds, omega-3 fatty acids constitute the most studied natural compounds for their potential beneficial role against BD progression and symptoms. This fact seems reasonable as BD has a low prevalence in the general population compared to other psychiatric diseases such as depression, anxiety, and stress. The low prevalence of BD does not permit us to take into consideration and examine a variety of potential risk factors that may be interrelated with the nutritional habits of BD patients.

Among the 40 retrieved clinical human studies, half (*n* = 21) of them were double-blind, placebo-controlled randomized clinical trials, which may derive more accurate and reliable evidence for the role of omega-3 fatty acids in reducing the severity of BD symptoms. However, most of the double-blind, placebo-controlled randomized clinical trials included a small sample size of BD patients and healthy controls, which was characterized by a low compliance rate and led to several volunteers abandoning the clinical trials. Moreover, most of the double-blind, placebo-controlled randomized clinical trials (*n* = 13) included BD patients without specifying what specific type of BD has been diagnosed in their enrolled patients. Only three double-blind, placebo-controlled randomized clinical trials included patients diagnosed with BD-I type. Another two double-blind, placebo-controlled randomized clinical trials included both BD-I and BD-II patients, two clinical trials included patients with bipolar spectrum disorder, and one clinical study included patients with BD not otherwise specified patients. Moreover, three double-blind, placebo-controlled randomized clinical trials included exclusively female BD patients, and three clinical trials included children and adolescent patients, while the remaining clinical studies contained adult patients. In view of the above analysis, there is a high heterogeneity concerning the study population of the double-blind, placebo-controlled randomized clinical trials, rendering their comparison quite difficult to draw accurate conclusions.

There are also certain cross-sectional clinical studies (*n* = 14) that cannot support causality effects and also include a small sample size with a low response rate. Among the cross-sectional studies, half (*n* = 8) included BD patients without specifying what specific type of BD has been diagnosed in their enrolled patients. Only two cross-sectional studies included exclusively patients diagnosed with BD-I, and another cross-sectional study included patients with bipolar spectrum disorder. Moreover, three cross-sectional studies included children and adolescent BD patients, while the remaining cross-sectional studies contained adult patients. Thus, there is also high heterogeneity concerning the study population of the cross-sectional studies. The cross-sectional studies did not require an omega-3 fatty acids dietary intervention, and thus, they could include a higher sample size of BD patients to increase their validity; however, they contain only a small number of enrolled patients. There is also only one prospective open-label clinical trial, including 20 children and adolescents with BD. Another type of clinical study determined the plasma or erythrocyte levels of EPA and/or DHA (*n* = 14) in association with the severity of BD symptoms. However, these studies also suffered from the above limitations.

**Figure 3:**
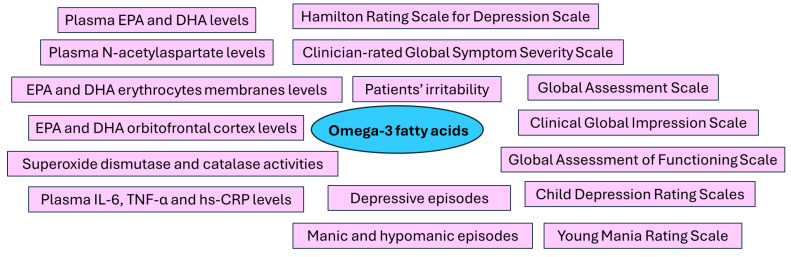
The main symptoms-related clinical diagnostic scales and the molecular biomarkers that may be affected by omega-3 fatty acids treatments.

Another important issue concerns whether the enrolled patients of the currently existing clinical trials received any medication treatment during the omega-3 fatty acids dietary intervention. In this respect, 14 clinical trials enrolled BD patients who did not receive any medication treatment (e.g., antipsychotics, mood stabilizers, anti-depressants, etc.) but received only omega-3 fatty acids in diverse doses for a rather short period (from 4 weeks to 6 months; median treatment duration: 12 weeks). Most of these studies did not show any significant effect of omega-3 fatty acids administration, and some of them showed a low or moderate beneficial effect. There were also nine clinical studies in which medication treatment was not available in order to draw more precise conclusions. On the other hand, there were 15 clinical studies in which omega-3 fatty acids were co-administrated with medication treatment. Most of these studies exhibited a potential positive effect against BD disorder symptomatology, supporting that omega-3 supplementary treatment in combination with the appropriate medication treatment may be more effective in attenuating the severity of BD symptoms. Collectively, there is a strong demand to perform clinical studies with a prospective design that will include an adequate number of BD patients. As prospective studies require much more time and follow-up setting and a higher number of qualified personnel expending a great deal of time and effort on such projects, the cross-sectional clinical study could initially be performed in a study population with a larger number of BD patients (e.g., more than 200 volunteers). Despite the heterogeneity of the existing evidence, it could be speculated that certain main symptoms-related clinical diagnostic scales and molecular biomarkers may be affected by omega-3 fatty acid treatments, as depicted in Figure 3.

### 4.2. Limitations

Due to the low prevalence of BD, a variety of risk factors should be taken into consideration in existing clinical studies. However, most of the studies did not adjust for potential differences in age and gender distributions as well as they did not control for low socioeconomic status, rural/urban ratios, marital status, alcoholism or smoking, and family history, which are well-known risk factors that predict the onset of BD. An additional concern is that variability in the definition and diagnosis of bipolar spectrum disorder and prevalence data across countries may vary by diagnostic instrument. A further limitation of the currently available clinical studies was that they could not determine if low seafood consumption increased lifetime risk for BD because of effects solely in adulthood or because of nutritional insufficiency in early neurological development or both. Although fish and seafood have been widely assessed for their beneficial effects on several chronic diseases, there is a lack of scientific evidence on BD.

While numerous studies show that omega-3 fatty acids can exert multiple beneficial effects, such as antioxidant and anti-inflammatory properties, which may regulate symptoms of BD, the clinical evidence remains inconsistent and, in many cases, insufficient. The existing results remain conflicting as some studies showed a positive effect in attenuating BD symptoms, whereas other studies did not find any effect. Most studies consisted of small sample sizes, lack of long-term follow-up, and variability in dosages and formulations of omega-3 fatty acids. Another serious concern is the fact that the enrolled patients in the currently available studies received different medication treatments for BD or did not receive any medication treatment during the clinical trials, as mentioned above. Thus, in some studies, omega-3 fatty acids were evaluated as monotherapy, whereas in other studies, they were explored as adjunctive treatment. In view of the above considerations, the comparison among the different clinical studies is difficult and not scientifically sound, and they cannot be generalized to the BD patient population. The above fact also renders the performance of meta-analysis studies insufficient and not reliable. Moreover, it is possible that omega-3 fatty acids may negatively interact with conventional BD medications, possibly resulting in adverse outcomes such as serotonin syndrome or exacerbation of manic symptoms. As a result, clinicians should monitor patients closely when using these supplements.

### 4.3. Future Perspectives

A promising research issue is that omega-3 fatty acids may be used as naturally occurring lead compounds by effectively applying recent developments in drug discovery processes. By effectively applying recent drug design technology, they could be synthesized and developed into more bioactive omega-3 fatty acids-related analogs with higher efficiency and target selectivity, reduced adverse side effects, and improved oral bioavailability. Machine learning methods have improved the domain of structure-based drug design in recent years, and they may be applied to omega-3 fatty acid derivatives [84]. Artificial intelligence methodologies to speed up and prevent failures in the drug design pipeline may also be utilized in the case of omega-3 fatty acids [85]. Intelligence techniques and quantum computation constitute another considerable advance in technology in generative chemistry and drug design developments, which researchers may manipulate in the case of omega-3 fatty acids [86]. Late-stage functionalization also offers new challenges for the exploitation of novel chemical moiety groups like omega-3 fatty acids and their future possible synthetic analogs across the end of a synthetic sequence that may promote new compounds, which may be quickly explored without laborious de novo chemical synthesis [87]. The above specific strategy could offer benefits such as efficient admission to various virtual-related libraries to investigate the structure-activity relationships and the improvement of physicochemical and pharmacokinetic properties of omega-3 fatty acids analogs [88,89]. Based on the conflicting results of the currently available studies, novel drug-like compounds should be synthesized using as a scaffold the chemical structure of the naturally occurring EPA and DHA omega-3 fatty acids.

Lipophilicity and biomimetic properties also exert crucial distinct and overlapping impacts in assisting the drug design process, especially in the case of bioavailability and pharmacokinetics [90]. Lipophilicity is unique in early drug design for library screening and for the identification of the most promising compounds to start with, while biomimetic properties are useful for the experimentally based evaluation of Absorption, Distribution, Metabolism, and Excretion (ADME) properties for the synthesized novel compounds, supporting the prioritization of drug candidates and guiding further synthesis. Lipophilicity also constitutes a vital physicochemical property in drug design as it relates to pharmacodynamic and pharmacokinetic properties as well as toxicological aspects of candidate drugs [91]. The above strategies are highly recommended for application to omega-3 fatty acids to render them more effective and specific to their target molecules [90,91].

By comprehensively reviewing the currently existing literature on the potential effect of omega-3 fatty acids against BD episodes and symptomatology, it remains questionable whether synthetic omega-3 fatty acids analogs beyond the naturally occurring EPA and DHA may also be effective against BD. In the last few years, several synthetic omega-3 fatty acids have been produced in order to be evaluated for their potential beneficial biological activities. Notably, resolvins, protectins, and maresins are a series of PUFA-derived molecules that exert important impacts on the resolution of inflammation [92]. They are termed specified proresolving mediators and assist in a return to homeostasis following an inflammatory response. These molecules are currently the focus of intensive investigation, primarily for their ability to suppress inflammation in chronic disease states, and therefore, they could be examined to determine whether they may attenuate BD episodes and symptoms [92]. More recently, OMT-28, a metabolically robust small molecule developed to mimic the structure and function of omega-3 epoxyeicosanoids, was found to exert potent cardio-protective and anti-inflammatory properties, supporting the assumption that regulating the bioavailability of omega-3 epoxyeicosanoids could improve their prospects as therapeutic agents [93]. In addition, Epeleuton (15 hydroxy eicosapentaenoic acid ethyl ester) is a novel, orally administered, second-generation omega-3 fatty acid with a favorable clinical safety profile, which found to prevent hypoxia/reoxygenation-induced activation of nuclear factor-κB with downregulation of the NLRP3 inflammasome in lung, kidney, and liver in a mouse model of sickle cell disease [94]. A metabolically robust synthetic analog of 17,18-epoxyeicosatetraenoic acid (EEQ-A) showed potent anti-inflammatory and immunomodulatory properties, which could support therapeutic strategies for ameliorating endotoxemia [95]. Overall, the above synthetic omega-3 fatty acids analogs could be tested for their action to attenuate the severity of BD symptoms and episodes. In this respect, taking EPA and/or DHA as lead compounds, medicinal chemists could synthesize in a laboratory setting novel omega-3 fatty acids analogs that may exert higher biological activity against the pathophysiological mechanism governing BD symptoms.

## 5. Conclusions

The clinical evidence concerning the effect of omega-3 fatty acids for the treatment of BD symptoms seems promising but remains rather insufficient and conflicting as more of the currently existing studies are characterized by several limitations. It is worth considering the possibility that an intervention could be more effective for prophylaxis (preventing episodes) than for relieving acute episodes or more effective in nonrapid cycling patients than in rapid-cycling patients. Thus, in spite of the less encouraging findings of these studies, it appears promising that additional studies are needed to assess the utility of omega-3 fatty acids in BD. Collectively, future research should focus on larger, well-controlled clinical trials to determine the efficacy of omega-3 fatty acids as supplement products for the treatment of BD, investigating potential interactions between them and conventional treatments and performing long-term studies to evaluate the efficiency and safety of these supplements in BD populations. Synthetic omega-3 fatty acids analogs should also be considered as potential therapeutic agents against BD symptomatology. Future clinical research should avoid the limitations of the currently existing clinical studies in order to draw more accurate conclusions.

## Data Availability

Data are available upon request from the corresponding author.

## References

[1] McIntyre R.S., Calabrese J.R. (2019). Bipolar depression: The clinical characteristics and unmet needs of a complex disorder. Curr. Med. Res. Opin..

[2] Collaborators GMD (2022). Global, regional, and national burden of 12 mental disorders in 204 countries and territories, 1990–2019: A systematic analysis for the Global Burden of Disease Study 2019. Lancet Psychiatry.

[3] Bonnín C.D.M., Reinares M., Martínez-Arán A., Jiménez E., Sánchez-Moreno J., Solé B., Montejo L., Vieta E. (2019). Improving Functioning, Quality of Life, and Well-being in Patients With Bipolar Disorder. Int. J. Neuropsychopharmacol..

[4] Moot W., Crowe M., Inder M., Eggleston K., Frampton C., Porter R.J. (2022). Domain-Based Functional Improvements in Bipolar Disorder After Interpersonal and Social Rhythm Therapy. Front. Psychiatry.

[5] Sanchez-Moreno J., Martinez-Aran A., Vieta E. (2017). Treatment of Functional Impairment in Patients with Bipolar Disorder. Curr. Psychiatry Rep..

[6] Depp C.A., Mausbach B.T., Harvey P.D., Bowie C.R., Wolyniec P.S., Thornquist M.H., Luke J.R., McGrath J.A., Pulver A.E., Patterson T.L. (2010). Social competence and observer-rated social functioning in bipolar disorder. Bipolar Disord..

[7] Lima I.M.M., Peckham A.D., Johnson S.L. (2018). Cognitive deficits in bipolar disorders: Implications for emotion. Clin. Psychol. Rev..

[8] GBD 2021 Forecasting Collaborators (2024). Burden of disease scenarios for 204 countries and territories, 2022–2050: A forecasting analysis for the Global Burden of Disease Study 2021. Lancet.

[9] Geddes J.R., Miklowitz D.J. (2013). Treatment of bipolar disorder. Lancet.

[10] Grande I., Berk M., Birmaher B., Vieta E. (2016). Bipolar disorder. Lancet.

[11] Berk M., Kapczinski F., Andreazza A.C., Dean O.M., Giorlando F., Maes M., Yücel M., Gama C.S., Dodd S., Dean B. (2011). Pathways underlying neuroprogression in bipolar disorder: Focus on inflammation, oxidative stress and neurotrophic factors. Neurosci. Biobehav. Rev..

[12] Muneer A. (2016). Bipolar Disorder: Role of Inflammation and the Development of Disease Biomarkers. Psychiatry Investig..

[13] Vega-Núñez A., Gómez-Sánchez-Lafuente C., Mayoral-Cleries F., Bordallo A., Rodríguez de Fonseca F., Suárez J., Guzmán-Parra J. (2022). Clinical Value of Inflammatory and Neurotrophic Biomarkers in Bipolar Disorder: A Systematic Review and Meta-Analysis. Biomedicines.

[14] Dzobo K. (2022). The Role of Natural Products as Sources of Therapeutic Agents for Innovative Drug Discovery. Compr. Pharmacol..

[15] Morris G., Walder K., Carvalho A.F., Tye S.J., Lucas K., Berk M., Maes M. (2018). The role of hypernitrosylation in the pathogenesis and pathophysiology of neuroprogressive diseases. Neurosci. Biobehav. Rev..

[16] Malhi G.S., Bassett D., Boyce P., Bryant R., Fitzgerald P.B., Fritz K., Hopwood M., Lyndon B., Mulder R., Murray G. (2015). Royal Australian and New Zealand College of Psychiatrists clinical practice guidelines for mood disorders. Aust. N. Z. J. Psychiatry.

[17] Mari J., Dieckmann L.H.J., Prates-Baldez D., Haddad M., Rodrigues da Silva N., Kapczinski F. (2024). The efficacy of valproate in acute mania, bipolar depression and maintenance therapy for bipolar disorder: An overview of systematic reviews with meta-analyses. BMJ Open.

[18] Nierenberg A.A., Agustini B., Köhler-Forsberg O., Cusin C., Katz D., Sylvia L.G., Peters A., Berk M. (2023). Diagnosis and Treatment of Bipolar Disorder: A Review. JAMA.

[19] Yatham L.N., Kennedy S.H., Parikh S.V., Schaffer A., Bond D.J., Frey B.N., Sharma V., Goldstein B.I., Rej S., Beaulieu S. (2018). Canadian Network for Mood and Anxiety Treatments (CANMAT) and International Society for Bipolar Disorders (ISBD) 2018 guidelines for the management of patients with bipolar disorder. Bipolar Disord..

[20] Chaachouay N., Zidane L. (2024). Plant-Derived Natural Products: A Source for Drug Discovery and Development. Drugs Drug Candidates.

[21] Bozzatello P., Novelli R., Montemagni C., Rocca P., Bellino S. (2024). Nutraceuticals in Psychiatric Disorders: A Systematic Review. Int. J. Mol. Sci..

[22] Ortega M.A., Álvarez-Mon M.A., García-Montero C., Fraile-Martínez Ó., Monserrat J., Martinez-Rozas L., Rodríguez-Jiménez R., Álvarez-Mon M., Lahera G. (2023). Microbiota–gut–brain axis mechanisms in the complex network of bipolar disorders: Potential clinical implications and translational opportunities. Mol. Psychiatry.

[23] Ashton M.M., Kavanagh B.E., Marx W., Berk M., Sarris J., Ng C.H., Hopwood M., Williams L.J., Dean O.M. (2021). A Systematic Review of Nutraceuticals for the Treatment of Bipolar Disorder. Can. J. Psychiatry.

[24] Bozzatello P., Brignolo E., De Grandi E., Bellino S. (2016). Supplementation with Omega-3 Fatty Acids in Psychiatric Disorders: A Review of Literature Data. J. Clin. Med..

[25] Nasir M., Bloch M.H. (2019). Trim the fat: The role of omega-3 fatty acids in psychopharmacology. Ther. Adv. Psychopharmacol..

[26] Saunders E.F., Ramsden C.E., Sherazy M.S., Gelenberg A.J., Davis J.M., Rapoport S.I. (2016). Omega-3 and Omega-6 Polyunsaturated Fatty Acids in Bipolar Disorder: A Review of Biomarker and Treatment Studies. J. Clin. Psychiatry.

[27] Lee-Okada H.C., Xue C., Yokomizo T. (2025). Recent advances on the physiological and pathophysiological roles of polyunsaturated fatty acids and their biosynthetic pathway. Biochim. Biophys. Acta Mol. Cell Biol. Lipids.

[28] Mischoulon D. (2018). Popular Herbal and Natural Remedies Used in Psychiatry. Focus.

[29] Sarris J., Moylan S., Camfield D.A., Pase M.P., Mischoulon D., Berk M., Jacka F.N., Schweitzer I. (2012). Complementary medicine, exercise, meditation, diet, and lifestyle modification for anxiety disorders: A review of current evidence. Evid. Based Complement. Altern. Med..

[30] Antao H.S., Sacadura-Leite E., Bandarra N.M., Figueira M.L. (2023). Omega-3 index as risk factor in psychiatric diseases: A narrative review. Front. Psychiatry.

[31] Rutkofsky I.H., Khan A.S., Sahito S., Kumar V. (2017). The Psychoneuroimmunological Role of Omega-3 Polyunsaturated Fatty Acids in Major Depressive Disorder and Bipolar Disorder. Adv. Mind Body Med..

[32] Stoll A.L., Severus W.E., Freeman M.P., Rueter S., Zboyan H.A., Diamond E., Cress K.K., Marangell L.B. (1999). Omega 3 fatty acids in bipolar disorder: A preliminary double-blind, placebo-controlled trial. Arch. Gen. Psychiatry.

[33] Chiu C.C., Huang S.Y., Su K.P., Lu M.L., Huang M.C., Chen C.C., Shen W.W. (2003). Polyunsaturated fatty acid deficit in patients with bipolar mania. Eur. Neuropsychopharmacol..

[34] Noaghiul S., Hibbeln J.R. (2003). Cross-national comparisons of seafood consumption and rates of bipolar disorders. Am. J. Psychiatry.

[35] Hirashima F., Parow A.M., Stoll A.L., Demopulos C.M., Damico K.E., Rohan M.L., Eskesen J.G., Zuo C.S., Cohen B.M., Renshaw P.F. (2004). Omega-3 fatty acid treatment and T(2) whole brain relaxation times in bipolar disorder. Am. J. Psychiatry.

[36] Osher Y., Bersudsky Y., Belmaker R.H. (2005). Omega-3 eicosapentaenoic acid in bipolar depression: Report of a small open-label study. J. Clin. Psychiatry.

[37] Sagduyu K., Dokucu M.E., Eddy B.A., Craigen G., Baldassano C.F., Yildiz A. (2005). Omega-3 fatty acids decreased irritability of patients with bipolar disorder in an add-on, open label study. Nutr. J..

[38] Keck P.E., Mintz J., McElroy S.L., Freeman M.P., Suppes T., Frye M.A., Altshuler L.L., Kupka R., Nolen W.A., Leverich G.S. (2006). Double-blind, randomized, placebo-controlled trials of ethyl-eicosapentanoate in the treatment of bipolar depression and rapid cycling bipolar disorder. Biol. Psychiatry.

[39] Frangou S., Lewis M., McCrone P. (2006). Efficacy of ethyl-eicosapentaenoic acid in bipolar depression: Randomised double-blind placebo-controlled study. Br. J. Psychiatry.

[40] Marangell L.B., Suppes T., Ketter T.A., Dennehy E.B., Zboyan H., Kertz B., Nierenberg A., Calabrese J., Wisniewski S.R., Sachs G. (2006). Omega-3 fatty acids in bipolar disorder: Clinical and research considerations. Prostaglandins Leukot. Essent. Fat. Acids.

[41] Frangou S., Lewis M., Wollard J., Simmons A. (2007). Preliminary in vivo evidence of increased N-acetyl-aspartate following eicosapentanoic acid treatment in patients with bipolar disorder. J. Psychopharmacol..

[42] Wozniak J., Biederman J., Mick E., Waxmonsky J., Hantsoo L., Best C., Cluette-Brown J.E., Laposata M. (2007). Omega-3 fatty acid monotherapy for pediatric bipolar disorder: A prospective open-label trial. Eur. Neuropsychopharmacol..

[43] Peet M., Horrobin D.F. (2002). A dose-ranging study of the effects of ethyl-eicosapentaenoate in patients with ongoing depression despite apparently adequate treatment with standard drugs. Arch. Gen. Psychiatry.

[44] Sublette M.E., Bosetti F., DeMar J.C., Ma K., Bell J.M., Fagin-Jones S., Russ M.J., Rapoport S.I. (2007). Plasma free polyunsaturated fatty acid levels are associated with symptom severity in acute mania. Bipolar Disord..

[45] Clayton E.H., Hanstock T.L., Hirneth S.J., Kable C.J., Garg M.L., Hazell P.L. (2008). Long-chain omega-3 polyunsaturated fatty acids in the blood of children and adolescents with juvenile bipolar disorder. Lipids.

[46] McNamara R.K., Jandacek R., Rider T., Tso P., Stanford K.E., Hahn C.G., Richtand N.M. (2008). Deficits in docosahexaenoic acid and associated elevations in the metabolism of arachidonic acid and saturated fatty acids in the postmortem orbitofrontal cortex of patients with bipolar disorder. Psychiatry Res..

[47] Clayton E.H., Hanstock T.L., Hirneth S.J., Kable C.J., Garg M.L., Hazell P.L. (2009). Reduced mania and depression in juvenile bipolar disorder associated with long-chain omega-3 polyunsaturated fatty acid supplementation. Eur. J. Clin. Nutr..

[48] McNamara R.K., Jandacek R., Rider T., Tso P., Dwivedi Y., Pandey G.N. (2010). Selective deficits in erythrocyte docosahexaenoic acid composition in adult patients with bipolar disorder and major depressive disorder. J. Affect. Disord..

[49] Qu Y., Chang L., Klaff J., Seemann R., Greenstein D., Rapoport S.I. (2006). Chronic fluoxetine upregulates arachidonic acid incorporation into the brain of unanesthetized rats. Eur. Neuropsychopharmacol..

[50] Raeder M.B., Fernø J., Glambek M., Stansberg C., Steen V.M. (2006). Antidepressant drugs activate SREBP and up-regulate cholesterol and fatty acid biosynthesis in human glial cells. Neurosci. Lett..

[51] Flock M.R., Skulas-Ray A.C., Harris W.S., Etherton T.D., Fleming J.A., Kris-Etherton P.M. (2013). Determinants of erythrocyte omega-3 fatty acid content in response to fish oil supplementation: A dose-response randomized controlled trial. J. Am. Heart Assoc..

[52] Sands S.A., Reid K.J., Windsor S.L., Harris W.S. (2005). The impact of age, body mass index, and fish intake on the EPA and DHA content of human erythrocytes. Lipids.

[53] Davison K.M., Kaplan B.J. (2012). Food intake and blood cholesterol levels of community-based adults with mood disorders. BMC Psychiatry.

[54] Jacka F.N., Pasco J.A., Mykletun A., Williams L.J., Nicholson G.C., Kotowicz M.A., Berk M. (2011). Diet quality in bipolar disorder in a population based sample of women. J. Affect. Disord..

[55] Kilbourne A.M., Rofey D.L., McCarthy J.F., Post E.P., Welsh D., Blow F.C. (2007). Nutrition and exercise behavior among patients with bipolar disorder. Bipolar Disord..

[56] Gracious B.L., Chirieac M.C., Costescu S., Finucane T.L., Youngstrom E.A., Hibbeln J.R. (2010). Randomized, placebo-controlled trial of flax oil in pediatric bipolar disorder. Bipolar Disord..

[57] Murphy B.L., Stoll A.L., Harris P.Q., Ravichandran C., Babb S.M., Carlezon W.A., Cohen B.M. (2012). Omega-3 fatty acid treatment, with or without cytidine, fails to show therapeutic properties in bipolar disorder: A double-blind, randomized add-on clinical trial. J. Clin. Psychopharmacol..

[58] Pomponi M., Janiri L., La Torre G., Di Stasio E., Di Nicola M., Mazza M., Martinotti G., Bria P., Lippa S., Natili R. (2013). Plasma levels of n-3 fatty acids in bipolar patients: Deficit restricted to DHA. J. Psychiatr. Res..

[59] Wozniak J., Faraone S.V., Chan J., Tarko L., Hernandez M., Davis J., Woodworth K.Y., Biedermanm J. (2015). A randomized clinical trial of high eicosapentaenoic acid omega-3 fatty acids and inositol as monotherapy and in combination in the treatment of pediatric bipolar spectrum disorders: A pilot study. J. Clin. Psychiatry.

[60] Fristad M.A., Young A.S., Vesco A.T., Nader E.S., Healy K.Z., Gardner W., Wolfson H.L., Arnold L.E. (2015). A Randomized Controlled Trial of Individual Family Psychoeducational Psychotherapy and Omega-3 Fatty Acids in Youth with Subsyndromal Bipolar Disorder. J. Child. Adolesc. Psychopharmacol..

[61] Saunders E.F., Reider A., Singh G., Gelenberg A.J., Rapoport S.I. (2015). Low unesterified:esterified eicosapentaenoic acid (EPA) plasma concentration ratio is associated with bipolar disorder episodes, and omega-3 plasma concentrations are altered by treatment. Bipolar Disord..

[62] Voggt A., Berger M., Obermeier M., Löw A., Seemueller F., Riedel M., Moeller H.J., Zimmermann R., Kirchberg F., Von Schacky C. (2015). Heart rate variability and Omega-3 Index in euthymic patients with bipolar disorders. Eur. Psychiatry.

[63] McNamara R.K., Jandacek R., Tso P., Blom T.J., Welge J.A., Strawn J.R., Adler C.M., DelBello M.P., Strakowski S.M. (2015). First-episode bipolar disorder is associated with erythrocyte membrane docosahexaenoic acid deficits: Dissociation from clinical response to lithium or quetiapine. Psychiatry Res..

[64] McNamara R.K., Jandacek R., Tso P., Blom T.J., Welge J.A., Strawn J.R., Adler C.M., Strakowski S.M., DelBello M.P. (2016). Adolescents with or at ultra-high risk for bipolar disorder exhibit erythrocyte docosahexaenoic acid and eicosapentaenoic acid deficits: A candidate prodromal risk biomarker. Early Interv. Psychiatry.

[65] Shakeri J., Khanegi M., Golshani S., Farnia V., Tatari F., Alikhani M., Nooripour R., Ghezelbash M.S. (2016). Effects of Omega-3 Supplement in the Treatment of Patients with Bipolar I Disorder. Int. J. Prev. Med..

[66] Wulsin L.R., Blom T.J., Durling M., Welge J.A., DelBello M.P., Adler C.M., McNamara R.K., Strakowski S.M. (2018). Cardiometabolic risks and omega-3 index in recent-onset bipolar I disorder. Bipolar Disord..

[67] Koga N., Ogura J., Yoshida F., Hattori K., Hori H., Aizawa E., Ishida I., Kunugi H. (2019). Altered polyunsaturated fatty acid levels in relation to proinflammatory cytokines, fatty acid desaturase genotype, and diet in bipolar disorder. Transl. Psychiatry.

[68] Ciappolino V., DelVecchio G., Prunas C., Andreella A., Finos L., Caletti E., Siri F., Mazzocchi A., Botturi A., Turolo S. (2020). The Effect of DHA Supplementation on Cognition in Patients with Bipolar Disorder: An Exploratory Randomized Control Trial. Nutrients.

[69] McNamara R.K., Strawn J.R., Tallman M.J., Welge J.A., Patino L.R., Blom T.J., DelBello M.P. (2020). Effects of Fish Oil Monotherapy on Depression and Prefrontal Neurochemistry in Adolescents at High Risk for Bipolar I Disorder: A 12-Week Placebo-Controlled Proton Magnetic Resonance Spectroscopy Trial. J. Child. Adolesc. Psychopharmacol..

[70] McPhilemy G., Byrne F., Waldron M., Hibbeln J.R., Davis J., McDonald C., Hallahan B. (2021). A 52-week prophylactic randomised control trial of omega-3 polyunsaturated fatty acids in bipolar disorder. Bipolar Disord..

[71] Saunders E.F.H., Mukherjee D., Myers T., Wasserman E., Hameed A., Bassappa Krishnamurthy V., MacIntosh B., Domenichiello A., Ramsden C.E., Wang M. (2022). Adjunctive dietary intervention for bipolar disorder: A randomized, controlled, parallel-group, modified double-blinded trial of a high n-3 plus low n-6 diet. Bipolar Disord..

[72] Wozniak J., Farrell A., DiSalvo M., Ceranoglu A., Uchida M., Vaudreuil C., Joshi G., Faraone S.V., Cook E., Biederman J. (2022). A Randomized, Double-Blind, Controlled Clinical Trial of Omega-3 Fatty Acids and Inositol as Monotherapies and in Combination for the Treatment of Pediatric Bipolar Spectrum Disorder in Children Age 5-12. Psychopharmacol. Bull..

[73] McNamara R.K., Chen C., Tallman M.J., Schurdak J.D., Patino L.R., Blom T.J., DelBello M.P. (2022). Familial risk for bipolar I disorder is associated with erythrocyte omega-3 polyunsaturated fatty acid deficits in youth with attention-deficit hyperactivity disorder. Psychiatry Res..

[74] Harsløf L.B., Larsen L.H., Ritz C., Hellgren L.I., Michaelsen K.F., Vogel U., Lauritzen L. (2013). FADS genotype and diet are important determinants of DHA status: A cross-sectional study in Danish infants. Am. J. Clin. Nutr..

[75] Koletzko B., Reischl E., Tanjung C., Gonzalez-Casanova I., Ramakrishnan U., Meldrum S., Simmer K., Heinrich J., Demmelmair H. (2019). FADS1 and FADS2 Polymorphisms Modulate Fatty Acid Metabolism and Dietary Impact on Health. Annu. Rev. Nutr..

[76] Berger M., Seemüller F., Voggt A., Obermeier M., Kirchberg F., Löw A., Riedel M., von Schacky C., Severus E. (2022). Omega-3 fatty acids in bipolar patients with a low omega-3 index and reduced heart rate variability: The “BIPO-3” trial. Int. J. Bipolar Disord..

[77] Zhang M., Li X., Dong L., Jin M., Xie M., Jia N., Liu Y., Xue F., Li W., Yang Y. (2023). Assessment of causal relationships between omega-3 and omega-6 polyunsaturated fatty acids in bipolar disorder: A 2-sample bidirectional mendelian randomization study. Food Funct..

[78] Eslahi H., Shakiba M., Saravani M., Payandeh A., Shahraki M. (2023). The effects of omega 3 fatty acids on the serum concentrations of pro inflammatory cytokines anddepression status in patients with bipolar disorder: A randomized double-blind controlled clinical trial. J. Res. Med. Sci..

[79] Gholipour D., Shahraki M., Saravani M., Payandeh A., Eslahi H. (2024). The Effect of Omega-3 Supplementation on Serum Levels of Antioxidant Status in Patients With Bipolar Disease: A Randomized Double-blind Controlled Clinical Trial. Basic. Clin. Neurosci..

[80] Zailani H., Wu S.K., Yang K.J., Malau I.A., Liao H.F., Chung Y.L., Chang J.P., Chiu W.C., Su K.P. (2024). Omega-3 polyunsaturated fatty acids in the prevention of relapse in patients with stable bipolar disorder: A 6-month pilot randomized controlled trial. Psychiatry Res..

[81] Li J., Hu R., Luo H., Guo Y., Zhang Z., Luo Q., Xia P. (2024). Associations between dietary habits and bipolar disorder: A diet-wide mendelian randomization study. Front. Psychiatry.

[82] Sarris J., Mischoulon D., Schweitzer I. (2012). Omega-3 for bipolar disorder: Meta-analyses of use in mania and bipolar depression. J. Clin. Psychiatry.

[83] Kishi T., Sakuma K., Okuya M., Ikeda M., Iwata N. (2021). Omega-3 fatty acids for treating residual depressive symptoms in adult patients with bipolar disorder: A systematic review and meta-analysis of double-blind randomized, placebo-controlled trials. Bipolar Disord..

[84] Turzo S.B.A., Hantz E.R., Lindert S. (2022). Applications of machine learning in computer-aided drug discovery. QRB Discov..

[85] Qureshi R., Irfan M., Gondal T.M., Khan S., Wu J., Hadi M.U., Heymach J., Le X., Yan H., Alam T. (2023). AI in drug discovery and its clinical relevance. Heliyon.

[86] Pyrkov A., Aliper A., Bezrukov D., Lin Y.C., Polykovskiy D., Kamya P., Ren F., Zhavoronkov A. (2023). Quantum computing for near-term applications in generative chemistry and drug discovery. Drug Discov. Toda.

[87] Montgomery A.P., Joyce J.M., Danon J.J., Kassiou M. (2023). An update on late-stage functionalization in today’s drug discovery. Expert. Opin. Drug Discov..

[88] Giaginis C., Tsantili-Kakoulidou A. (2013). Quantitative Structure -Retention Relationships as a useful tool to characterize chromatographic systems and their potential to simulate biological processes. Chromatographia.

[89] Vallianatou T., Giaginis C., Tsantili-Kakoulidou A. (2015). The impact of physicochemical and molecular properties in drug design: Navigation in the “drug-like” chemical space. Adv. Exp. Med. Biol..

[90] Tsopelas F., Giaginis C., Tsantili-Kakoulidou A. (2017). Lipophilicity and biomimetic properties to support drug discovery. Expert. Opin. Drug Discov..

[91] Giaginis C., Tsopelas F., Tsantili-Kakoulidou A. (2018). The Impact of Lipophilicity in Drug Discovery: Rapid Measurements by Means of Reversed-Phase HPLC. Methods Mol. Biol..

[92] Daly K., O’Sullivan K., O’Sullivan T.P. (2022). Major structure-activity relationships of resolvins, protectins, maresins and their analogues. Future Med. Chem..

[93] Kranrod J., Konkel A., Valencia R., Darwesh A.M., Fischer R., Schunck W.H., Seubert J.M. (2024). Cardioprotective properties of OMT-28, a synthetic analog of omega-3 epoxyeicosanoids. J. Biol. Chem..

[94] Mattè A., Federti E., Recchiuti A., Hamza M., Ferri G., Riccardi V., Ceolan J., Passarini A., Mazzi F., Siciliano A. (2024). Epeleuton, a novel synthetic ω-3 fatty acid, reduces hypoxia/reperfusion stress in a mouse model of sickle cell disease. Haematologica.

[95] Shikuma A., Kami D., Maeda R., Suzuki Y., Sano A., Taya T., Ogata T., Konkel A., Matoba S., Schunck W.H. (2022). Amelioration of Endotoxemia by a Synthetic Analog of Omega-3 Epoxyeicosanoids. Front. Immunol..

